# Holmium-Containing Metal-Organic Frameworks as Modifiers for PEBA-Based Membranes

**DOI:** 10.3390/polym15183834

**Published:** 2023-09-20

**Authors:** Anna Kuzminova, Mariia Dmitrenko, Kirill Salomatin, Olga Vezo, Sergey Kirichenko, Semyon Egorov, Marina Bezrukova, Anna Karyakina, Alexey Eremin, Ekaterina Popova, Anastasia Penkova, Artem Selyutin

**Affiliations:** 1 Saint-Petersburg State University, 7/9 Universitetskaya Emb., St. Petersburg 199034, Russia; a.kuzminova@spbu.ru (A.K.); m.dmitrienko@spbu.ru (M.D.); st106790@student.spbu.ru (K.S.); o.vezo@spbu.ru (O.V.); sergey.kirichenko@spbu.ru (S.K.); alar.zeee@gmail.com (S.E.); yara_2000@mail.ru (A.K.); a.penkova@spbu.ru (A.P.); 2 Institute of Macromolecular Compounds, Russian Academy of Sciences, 31 Bolshoy pr., St. Petersburg 199004, Russia; bezrukova@imc.macro.ru (M.B.); ave@hq.macro.ru (A.E.); ekaterinaapopova@yandex.ru (E.P.); 3 Faculty of Chemical and Biotechnology, Organic Chemistry Department, Saint-Petersburg State Institute of Technology (Technical University), 24-26/49 Letter A Moskovski Ave., St. Petersburg 190013, Russia; 4 Faculty of Industrial Drug Technologies, Department of Chemical Technology of Medicinal Substances, Saint-Petersburg State Chemical and Pharmaceutical University, 14 Prof. Popova Str., St. Petersburg 197022, Russia

**Keywords:** MOF, MOF-polymer composites, polyether block amide, mixed matrix membrane, dye filtration

## Abstract

Recently, there has been an active search for new modifiers to create hybrid polymeric materials for various applications, in particular, membrane technology. One of the topical modifiers is metal-organic frameworks (MOFs), which can significantly alter the characteristics of obtained mixed matrix membranes (MMMs). In this work, new holmium-based MOFs (Ho-MOFs) were synthesized for polyether block amide (PEBA) modification to develop novel MMMs with improved properties. The study of Ho-MOFs, polymers and membranes was carried out by methods of X-ray phase analysis, scanning electron and atomic force microscopies, Fourier transform infrared spectroscopy, low-temperature nitrogen adsorption, dynamic and kinematic viscosity, static and dynamic light scattering, gel permeation chromatography, thermogravimetric analysis and contact angle measurements. Synthesized Ho-MOFs had different X-ray structures, particle forms and sizes depending on the ligand used. To study the effect of Ho-MOF modifier on membrane transport properties, PEBA/Ho-MOFs membrane retention capacity was evaluated in vacuum fourth-stage filtration for dye removal (Congo Red, Fuchsin, Glycine thymol blue, Methylene blue, Eriochrome Black T). Modified membranes demonstrated improved flux and rejection coefficients for dyes containing amino groups: Congo Red, Fuchsin (PEBA/Ho-1,3,5-H_3_btc membrane possessed optimal properties: 81% and 68% rejection coefficients for Congo Red and Fuchsin filtration, respectively, and 0.7 L/(m^2^s) flux).

## 1. Introduction

The properties of metal-organic frameworks (MOFs) are related to the variety of possible structures. Their structure depends not only on the properties of the metal nodes and organic linkers but also on the preparation route and synthesis conditions—temperature, solvent, mixing, etc. In recent years, the interest of researchers in holmium-containing MOFs as multifunctional systems has been actively growing [1]. The multifunctionality of such compounds [2] is associated with good catalytic activity [3,4,5,6,7,8], their magnetic [9,10,11,12] and luminescent properties [13], as well as the possibility of using them in various therapies [14]. Many researchers are now actively looking for ways to obtain new holmium-based frameworks [15], and various studies have been carried out based on known structures [16,17]. More recently, there has been a move away from the typical solvents used in MOF preparation, such as dimethylformamide (DMF) [18,19,20,21,22,23]. In this work, ethanol was chosen as a “green” solvent for the synthesis of Ho-based MOFs (Ho-MOFs) with different ligands. Previously, ethanol has already been shown to be a promising solvent for the synthesis of other MOFs [22,24,25,26,27,28]. To the best of our knowledge, there is little information on the application of this solvent for the synthesis of Ho-MOFs. It should also be mentioned that the use of Ho nitrates in an ethanol media during the decomposition at elevated temperatures led to reduced formation of nitrogen oxides by recovering them to gaseous nitrogen (stable and harmless compound) [29,30,31]. Ethanol is able to deprotonate the carboxyl groups of linkers (benzoic acids) used in this work, which is the main stage in the mechanism of self-assembly of metal-organic frameworks. While using DMF, such a proton transfer at elevated temperature resulted in the formation of various biologically active amines (potential carcinogenic agents).

Membranes and thin solid films with metal ions [32,33,34] have been used in many fields, such as seawater desalination, wastewater treatment and gas separation [35,36,37]. Most modern research into the use of membrane technologies for the separation of components is based on the use of mixed matrix membranes (MMMs) containing various additives, such as metal oxides, fullerene and its derivatives, MOFs, etc. [38,39,40,41,42,43,44]. MOFs are one of the promising modifiers of membranes due to their surface area and functionality, controlled size and form of pores and particles [45]. MMMs based on polymer/MOFs are actively used for different membrane processes—pervaporation, ultrafiltration, gas separation and nanofiltration [41,42,46,47,48]. In recent years, nanofiltration MMMs based on polymer/MOFs were investigated for metal ion [42,46,49] and dye [49,50,51,52] removal from water. In the study [49], polyethersulfone (PES) membranes modified with various UiO-66-NH_2_ content were tested in the nanofiltration of three dyes (Methylene Blue, Rhodamine B and Reactive Blue 21). The membrane with 0.01 wt% UiO-66-NH_2_ exhibited the best performance for the removal of dyes: rejection coefficient of 95.8% Methylene Blue, 94.2% Rhodamine B and 83.6% Reactive Blue 21. Modified metal-organic framework nanoparticles (MOF@Fe_3_O_4_) were used for the modification of porous PES membranes for Acid Orange 7 removal from water [50]. It was found that 0.5 wt% MOF@Fe_3_O_4_ in the PES matrix was the optimal concentration and led to membrane-improved water flux (28.5 kg/(m^2^h)) due to increased surface hydrophilicity, MOF pore size and void spaces. With regards to increasing the efficiency of dye removal (88.3% Acid Orange 7) under optimal conditions (pH = 3), the authors explained that this is due to the repulsive force between the positively charged dye (due to the presence of azo- and hydroxyl functional groups) and the positively charged modified membrane [50]. Membranes based on polyethyleneimine (PEI) modified with new MOF BUT-203 were developed and investigated in nanofiltration for the removal of anionic dyes (Methyl blue, Congo Red, Acid Fuchsin, Eriochrome Black T and Acid Orange II) [51]. The addition of BUT-203 nanosheets into the PEI membrane led to improved separation performance and stability. Membranes modified by 73% BUT-203 had optimal properties: water permeance up to 870 L/(m^2^ h MPa) and rejection coefficients of 97.9% for Methyl blue, 99.9% for Congo Red, 97.9% for Acid Fuchsin, 99.7% for Eriochrome Black and 93.7% for Acid Orange II. In the study [52], the highly hydrolytic stable MOF BUT-8(A) was used as a modifier for the creation of nanofiltration MMMs based on PEI for dye removal from water. The high separation performance of modified membranes suggests a promising application for water purification. It was found that the modified membrane with a BUT-8(A) loading (50 wt%) had optimal transport characteristics: high water permeances (396–683 L/(m^2^ h MPa)) and rejection coefficients of 98.3% for Methyl blue, 99.8% for Congo Red, 89.3% for Acid Fuchsin, 82.1% for Methyl Orange. High water flux and separation mechanisms were attributed to the facilitated molecule transportation through the channels of BUT-8(A). To the best of our knowledge, there is no information on the study of membranes based on PEBA modified with Ho-MOFs for the removal of dyes from water.

Polyester block amides (PEBA) or Pebax^®^ elastomers (trade name) are block copolymers made up of rigid polyamide blocks, soft polyether blocks and plasticizer-free thermoplastic elastomers (TPEs) [53]. These polymers have a number of properties, such as flexibility, chemical resistance and strength, that make them promising for use in membrane technologies [54,55,56]. Recently, a number of studies have been carried out on the use of mixed matrix membranes based on PEBA not only for the separation of gases [57,58,59,60,61,62,63,64,65,66] but also for the extraction of various biofuel components [67,68], alcohols [69,70], desulphurization [71], alcohol dehydration [72], esterification [73] and pervaporation [74,75]. The high degree of combinatoriality in the preparation of block copolymers makes it possible to obtain a large number of commercially available polymers with different compositions and properties [76]. To improve the transport properties, PEBA-based membranes are modified with various particles such as ZnO [77,78], chitosan-wrapped multiwalled carbon nanotubes (CWNTs) [79], graphene [80], Aminosilane-Functionalized Zeolite Y [66], modified multi-walled carbon nanotubes (MWCNTs) [81], polyethylene glycol (PEG-400) and TiO_2_ [82], covalent organic frameworks (COFs) [83], MOFs [84,85,86,87], etc. Particular attention should be paid to MOFs due to their unique characteristics; however, PEBA/MOF membranes are developed mainly for diffusion membrane processes (pervaporation [84,85] and gas separation [86,87]).

The novelty of this work consists of (i) the use of new ligands (1,2,4-H_3_btc, 1,2-H_2_bdc and 1,3-H_2_bdc) and an atypical solvent—ethanol (to avoid the formation of toxic impurities) for synthesis Ho- MOFs, (ii) the application of synthesized Ho-MOFs for the preparation of MMMs based on PEBA. The obtained MOFs and MMMs based on PEBA/Ho-MOFs composites were investigated by various analysis methods. The study of the dependence of the molecular weight and viscosity for the commercial PEBA Pebax-2533 solutions was also carried out to determine polymer properties for obtaining MMMs with tailored characteristics. The possibility of using membrane PEBA/Ho-MOFs materials to remove various dyes (Congo Red, Fuchsin, Glycine thymol blue, Methylene blue, Eriochrome Black T) from aqueous solutions in the process of vacuum filtration was first studied.

## 2. Materials and Methods

### 2.1. Materials

The following reagents were used in this work to obtain polymer composite membrane materials: 1,3,5-H_3_btc—trimesic acid; 1,2,4-H_3_btc—trimelic acid; 1,2-H_2_bdc—phthalic acid; 1,3-H_2_bdc—isophthalic acid; 1,4-H_2_bdc—terephthalic acid (structures in Figure S1 of Supplementary Materials); ethanol; tetrahydrofuran; 1-butanol; Ho(NO_3_)_3_·5H_2_O. Congo Red, Fuchsin, Glycine thymol blue, Methylene blue and Eriochrome Black T (Vecton, St. Petersburg, Russia) were used as dyes. All of the linkers and dyes (structural formula, molecular weight, maximum absorption wavelength in Table S1 of Supplementary Materials) were of analytical grade and used without further purification. Pebax-2533 (containing approximately 80 wt.% polyether segments and 20 wt.% polyamide segments) was purchased from Hebei Luozheng Technology Co., Ltd. (Shijiazhuang, China). Distillation at atmospheric pressure was used to purify the ethanol and 1-butanol. To purify tetrahydrofuran, peroxides were removed by treatment with aqueous ferrous sulfate followed by solid KOH. The solvent was then dried and fractionally distilled from sodium [88].

### 2.2. Ho-MOFs Preparation

For the solvothermal synthesis of Ho-MOFs, a solution of holmium nitrate and the corresponding benzenecarboxylic acid in ethanol was placed in a steel autoclave with a Teflon liner (25 mL). For 0.5000 g (1.134 mmol) Ho(NO_3_)_3_·5H_2_O—1.134 mmol (0.2382 g) of 1,3,5-H_3_btc and 1,2,4-H_3_btc; 1.700 mmol (0.2825 g) of 1.2-H_2_bdc, 1,3-H_2_bdc and 1,4-H_2_bdc were introduced into reaction mixture. pH values were <7. Each autoclave was placed in an oven where it was kept for 130 h at a temperature of 150 °C. After cooling to room temperature, the resulting light-yellow precipitates were centrifuged at 5000 rpm, and the precipitates were separated and washed with fresh ethanol. The washing procedure was repeated three times. The samples obtained were then washed twice with methanol and dichloromethane in an ultrasonic bath, separated from the washing solvents, dried and activated in a vacuum oven (residual pressure 20 mm Hg) at a temperature of 150 °C for 65 h.

### 2.3. Ho-MOFs Investigation

The phase composition and X-ray crystal structure of the resulting Ho-based MOFs were determined using a MiniFlexII powder diffractometer (Rigaku, Japan) with CuKα-radiation.

Physisorption measurements with nitrogen were performed at 77 K with an ASAP 2020 MP analyzer (Micromeritics, Norcross, GA, USA). The specific surface area for the obtained MOFs was calculated through the BET method [89]. Determination of the pore size distribution was achieved by the BJH method with Harkins–Jura and Faas correction [90,91].

The structural changes of the Ho-MOFs were investigated by Fourier-transform infrared spectroscopy (FTIR) using an IRAffinity-1S spectrometer (Shimadzu, St. Petersburg, Russia) and an attenuated total reflectance accessory (PIKE Technologies, St. Petersburg, Russia) in the range of 400–4000 cm^−1^ at 25 °C in KBr matrix.

The morphology of the developed Ho-MOFs was studied by scanning electron microscopy (SEM) using a Zeiss Merlin SEM (Carl Zeiss SMT, Oberkochen, Germany) at an accelerating voltage of 1 kV and electron beam current of 100 pA to prevent surface charging.

### 2.4. PEBA Investigation

The molar mass and dispersion of the PEBA polymer were determined using gel permeation chromatography on an LC-20AD liquid chromatograph with a SIL-20AC autosampler, a DGU-20A3 degasser and an RID-20A refractive index detector (Shimadzu). Separation was performed using a PSS SDV precolumn (50 × 8 mm, 5 µm) and PSS SDV columns (300 × 8 mm, 5 µm, PSS) with a pore size of 1000 and 1,000,000 Å. The tetrahydrofuran was eluent. The incubator temperature was 40 °C (CTO-20AC (Shimadzu)), the injection volume was 100 µL, and the eluent flow rate was 1 mL/min. The calibration dependence was built using narrowly dispersed polymer standards—samples of polystyrenes with a molecular weight of 19,700–2,520,000 Da. Software PSS Win GPC UniChrom (v 8.33 Build 9050) was applied.

The dynamic and kinematic viscosity of the 1-butanol-PEBA fluids was determined using the LOVIS 2000 M microviscometer. The determination of the mass distribution of a polymer in 1-butanol was carried out at the Photocor Complex facility (Photocor, Moscow, Russia). A 2 wt% solution of PEBA in 1-butanol was used as the initial sample. The thermostat temperature was (298.15 ± 0.05) K. The measurements were carried out with a light source wavelength of λo = 445 nm (a semiconductor laser with a radiant power of 25 mW). The intensity of the light scattered in the solutions was measured in the range of scattering angles θ from 40° to 140° after 10°. The intensity of the scattered light is proportional to r3, and the smaller the scattering angle, the greater the contribution of large particles to the scattering, leading to a distortion of the scattering indicatrix. Therefore, when analyzing static light scattering data, only those angles are used for which there is no significant deviation from the linear dependence Hc/R(θ).

The Zimm double extrapolation method [92] was used to calculate the Mw. The excess light scattering intensity I(q,c) with respect to the solvent in dilute polymer solutions is expressed by Equation (1):

(1) $$\frac{Hc}{R(q,\;c)}=\frac{1}{M_{w}P\left(\theta \right)}+2A_{2}c,$$

where *c* is the polymer concentration, *q = (4πn*_o_*/λ*_o_*)sin(θ/2)* is the scattering wave vector, *A*_2_ is the second virial coefficient, *H* = 4π^2^*n*_0_^2^(*dn/dc*)^2^/N_A_λ_0_^4^ is the scattering constant, *n*_0_ is the refractive index of the solvent, *dn/dc* is the refractive index increment of the polymer solution and *N_A_* is the Avogadro number. According to the Zimm approximation, the function $P\left(\theta \right)=\left(1-\frac{1}{3}q^{2}R_{g}^{2}\right)$ is the particle form factor, which allows the radius of gyration *R_g_* to be determined.

From the total intensity of the light scattered by the solution, it is necessary to subtract the intensity of the light scattered by the solvent. This gives the final expression for the angular dependence of the Rayleigh coefficient (2):

(2) $$R\left(\theta \right)=\frac{\left(I\left(\theta \right)-I_{0}\left(\theta \right)\right)}{I_{t}\left(\theta \right)}R_{t}\frac{n_{0}}{n_{t}},$$

where *I*(θ) is the intensity of light scattered by the polymer solution, *I_0_*(θ) is the intensity of light scattered by the solvent (water), *I_t_*(θ) is the intensity of light scattered by the standard solvent (toluene), *n_0_* is the refractive index of the solvent and *n_t_* is the refractive index of the standard solvent (toluene). Rt = 60.35 × 10^−6^ cm^−1^ (λ = 445 nm, T = 298.15 K) [93]. Autocorrelation functions in solutions were measured in the range of scattering angles θ from 30° to 140°. The scattered light is received by a photodetector. The signal from the photodetector output is processed by a digital correlator. From the autocorrelation function obtained, the *DynaLS* program calculates the characteristic relaxation time of the fluctuations τ, the mean size or size distribution of the dispersed particles.

### 2.5. Dense Membrane Preparation

For the preparation of dense mixed matrix membranes based on PEBA, all components were placed in a glass test tube. 1-butanol was added to the MOF and polymer. Dissolution of the polymer in solvent and dispersion of the MOF particles were carried out using an ultrasonic bath at a temperature of 60 °C with alternating stirring on a vortex. The resulting viscous dispersion was poured into a Petri dish and dried in an oven at 60 °C for 24 h. The resulting composite membrane was separated from the dish while soaking in water.

### 2.6. Membrane Investigation

A BRUKER-TENSOR 27 Spectrometer (Bruker, Ettlingen, Germany) with an attenuated total reflectance (ATR) accessory was used to determine the structure of PEBA and PEBA/Ho-MOFs membranes in the range 600–4000 cm^−1^ at ambient temperature.

The thermochemical properties of the PEBA and PEBA/Ho-MOFs membranes were studied by thermogravimetric analysis (TGA) using Thermobalance TG 209 F1 Libra (Netzsch, Leuna, Germany) with the heating temperature range in the range 37–570 °C and the heating rate of 10 °C/min in an argon atmosphere.

The cross-section of the PEBA and PEBA/Ho-MOFs membranes was investigated by scanning electron microscopy (SEM) using Zeiss Crossbeam 1540XB (Carl Zeiss SMT, Oberkochen, Germany) at 5 kV. A cross-section of the membranes was obtained by breaking the membrane in liquid nitrogen perpendicular to the surface.

The surface topography of the PEBA and PEBA/Ho-MOFs membranes was studied by atomic force microscopy (AFM) using an NT-MDT NTegra Maximus atomic force microscope (NT-MDT Spectrum Instruments, Moscow, Russia) with standard silicon cantilevers and rigidity of 15 N·m^−1^ in tapping mode.

Changes in the surface hydrophilic/hydrophobic balance of PEBA and PEBA/Ho-MOFs membranes were studied by measuring the contact angles. The sessile drop method was used, which consisted of applying a drop of water to the surface of the membrane. Measurements were taken on the Goniometer LK-1 instrument (NPK Open Science Ltd., Krasnogorsk, Russia), and the “DropShape” software was used to analyze the results.

To study the retention capacity of the membranes, the dye solution was filtered through the PEBA and PEBA/Ho-MOF membrane using a vacuum filtration system with a water jet pump. Congo Red, Fuchsin, Glycine thymol blue, Methylene blue and Eriochrome Black T were used as dyes. An initial solution of dye was prepared in distilled water at a concentration of 0.1% by weight. The resulting filtrate was analyzed for dye content by optical spectrophotometry in the SF-102 spectrophotometer (JSC “Aquilon”, St. Petersburg, Russia), and wavelengths of absorbed light are presented in Table S1. The resulting filtrate was also subjected to filtration through the same membrane. Filtration was continued until the filtered dye solution was halved. After filtering the Congo Red dye solution four times under reduced pressure and determining the residual concentrations, the dye rejection coefficient was determined using Equation (3):

(3) $$R=\left(1-\frac{C_{f}}{C_{0}}\right)*100\%,$$

where *R* is the rejection coefficient, and *C_f_* and *C_o_* are the concentrations of Congo Red dye in the filtrate and initial solution, respectively.

To calculate the flux (*J*) of the membranes, Equation (4) was used [94]:

(4) $$J=\frac{V}{A\cdot t}$$

where *V* (L) is the permeate volume, *A* (3.14 × 10^−4^ m^2^) is the effective area of the membrane and *t* is the time of the measurement (s).

## 3. Results and Discussions

### 3.1. Ho-MOFs Investigation

**Figure 1:**
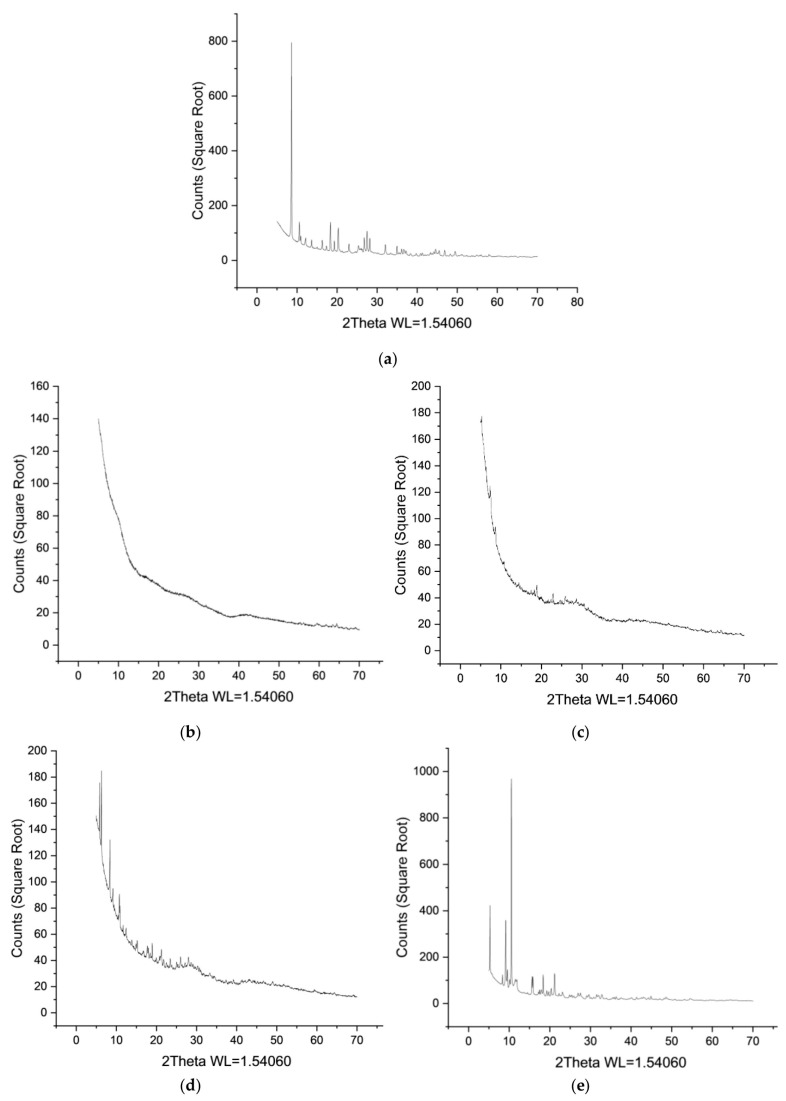
X-ray powder diffraction patterns for Ho-MOFs: (**a**) Ho-1,3,5-H_3_btc; (**b**) Ho-1,2,4-H_3_btc; (**c**) Ho-1,2-H_2_bdc; (**d**) Ho-1,3-H_2_bdc; (**e**) Ho-1,4-H_2_bdc.

The resulting Ho-MOFs were studied by X-ray phase analysis, scanning electron microscopy, FTIR spectroscopy and low-temperature nitrogen adsorption after synthesis, washing and thermal activation in a vacuum oven. The X-ray diffraction patterns are shown in Figure 1.

In Figure 1, Ho-1,2,4-H_3_btc (Figure 1b) and Ho-1,2-H_2_bdc MOFs (Figure 1c) are typical of the X-ray amorphous state. Due to the low signal intensity, the X-ray structures of these synthesized MOFs could not be determined from the diffraction patterns. For all other materials Ho-1,3,5-H_3_btc, Ho-1,3-H_2_bdc, Ho-1,4-H_2_bdc (Figure 1a,d,e), reflections of different X-ray crystal structures were observed in the diffraction patterns. These Ho-MOFs X-ray structures were additionally analyzed by matching diffractograms in the Cambridge crystallographic database and presented in Figure S2 of Supplementary Materials. It demonstrates the comparison of the X-ray powder diffraction pattern of the synthesized Ho-1,4-H_2_bdc with the diffraction pattern simulated from the *cif*-file for Y-1,4-H_2_bdc MOF [95]. The shift along the abscissa is related to the difference in the parameters of the crystal cells. This, in turn, is due to the difference in the crystal radii of the metal atoms. The powder diffraction patterns of Ho-1,3-H_2_bdc are similar to those of Al-1,3-H_2_bdc MOF [96]. The X-ray structure already known for holmium trimesate [97] was obtained by synthesis from holmium nitrate and trimesic acid in DMF/ethanol/water mixture. The X-ray structures of resulting MOFs are shown in Figure S3 of Supplementary Materials.

**Figure 2:**
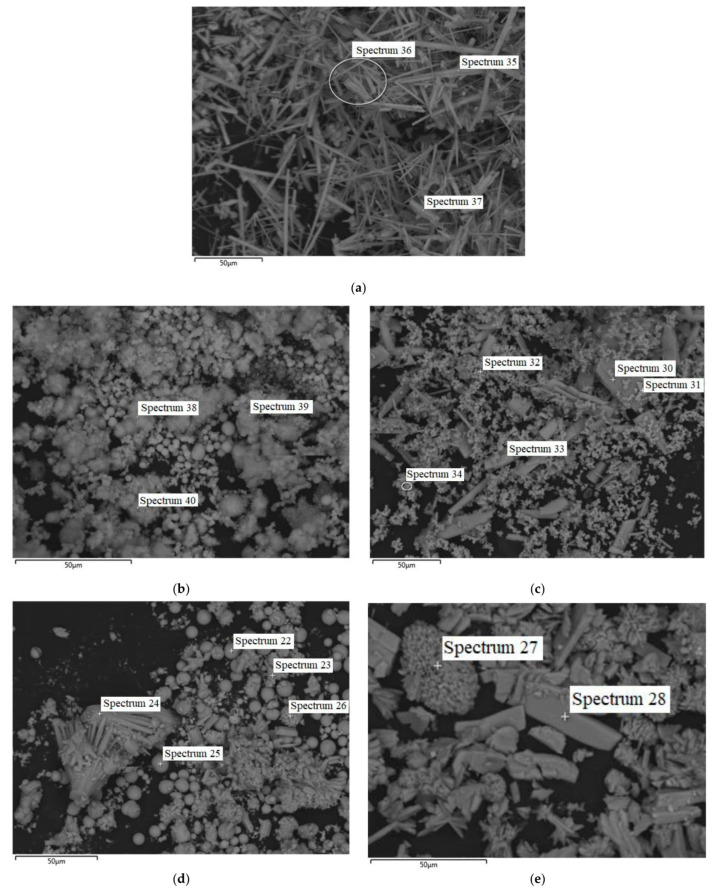
SEM micrographs for Ho-MOFs: (**a**) Ho-1,3,5-H_3_btc; (**b**) Ho-1,2,4-H_3_btc; (**c**) Ho-1,2-H_2_bdc; (**d**) Ho-1,3-H_2_bdc; (**e**) Ho-1,4-H_2_bdc.

Scanning electron microscopy was carried out with additional energy-dispersive elemental analysis. SEM images of the Ho-MOFs obtained with spectra numbers and locations for elemental analysis (presented in Figures S4–S8 of Supplementary Materials) are shown in Figure 2.

The habit of the crystals for the obtained Ho-1,3,5-H_3_btc corresponds to the crystal structure with long needle-shaped crystals (Figure 2a). In the other micrographs, the morphology of the crystals of the Ho-MOFs obtained is poorly defined: it is possible to distinguish between various intergrowths of particles and unformed non-crystalline structures.

Low-temperature nitrogen adsorption–desorption isotherms show different behavior of the obtained Ho-MOFs (Figure S9 of Supplementary Materials). Table 1 shows the values of the specific surface area for Ho-MOFs calculated based on obtained data.

The shape of the nitrogen adsorption isotherms (Figure S9 of Supplementary Materials) indicates the production of microporous materials. To confirm the porous structure of MOFs, their specific surface areas were calculated through the BET method. It was demonstrated that for Ho-1,2-H_2_bdc, the high specific surface area value (241 m^2^/g) was noted. For the rest of the Ho-MOFs, low specific surface area values were observed. The difference in data dependencies for the Ho-MOFs indicated the production of a very highly porous substance. However, it should be noted that the specific surface area cannot be calculated by the BET method quantitatively; only a qualitative comparison of the studied samples can be carried out. This is due to the fact that developed Ho-MOFs are “breathable” because they absorb gases from the atmosphere well. During sample preparation for measuring the specific surface area, a large number of pores are occupied and not available for the absorbate gas (nitrogen). Thus, the resulting values of the specific surface area were approximately estimated. For gas separation, a large specific surface plays a major role, but for processes of liquid mixture separation, it is not so significant, especially taking into account the fact that MOFs are introduced inside the PEBA polymer film in this study. The habit and size of crystals, as well as the porosity of MOFs, will largely determine the transport properties of MMMs [42,46,98]. It should be noted that in order to obtain highly porous materials, many factors must be taken into account, in particular, the used solvent during synthesis. Normally, DMF is used to obtain MOFs, but the synthesis in this work was carried out in ethanol. This led to the formation of coordination frameworks in which the solvent molecules were much more strongly bound to the holmium node. However, the ethanol molecules coordinated to the holmium atom in the structure are mobile due to rotational degrees of freedom. Therefore, the resulting MOF can be attributed to so-called “breathing”, which makes it possible to have a material with a highly developed pore space [99,100,101,102].

**Figure 3:**
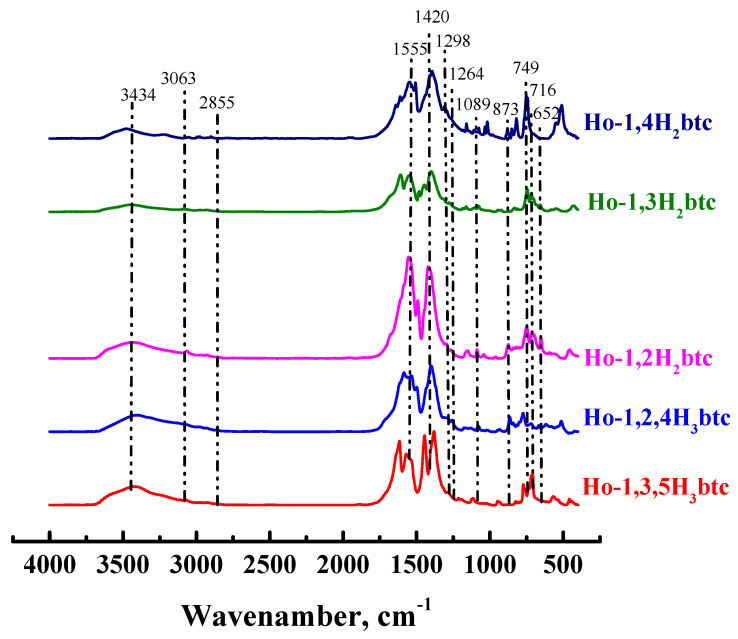
IR spectra of Ho-MOFs.

In the IR spectra (Figure 3), a broad peak at 3434 cm^−1^ is a characteristic peak of the -OH group. The absorption at 1555 cm^−1^ is associated with stretching of C=O groups, and at 1420 cm^−1^—with asymmetric stretching of O–C–O, belonging to the carboxyl group of the ligands; symmetric and asymmetric stretching of C–H manifests itself in the range of 2855–3063 cm^−1^; stretching of the same group appears at 1420 cm^−1^. However, the peaks at 1089, 873 and 749 cm^−1^ are due to -CH_3_ stretching. Furthermore, two sharp peaks at 716 and 652 cm^−1^ are due to vibrations of holmium nodes surrounded by oxygen atoms of the carboxylic groups of the linker and the hydroxyl group of ethanol. For many MOFs, similar values of the vibrational energy of a metal node have been determined [17,103,104,105].

### 3.2. PEBA Investigation

**Figure 4:**
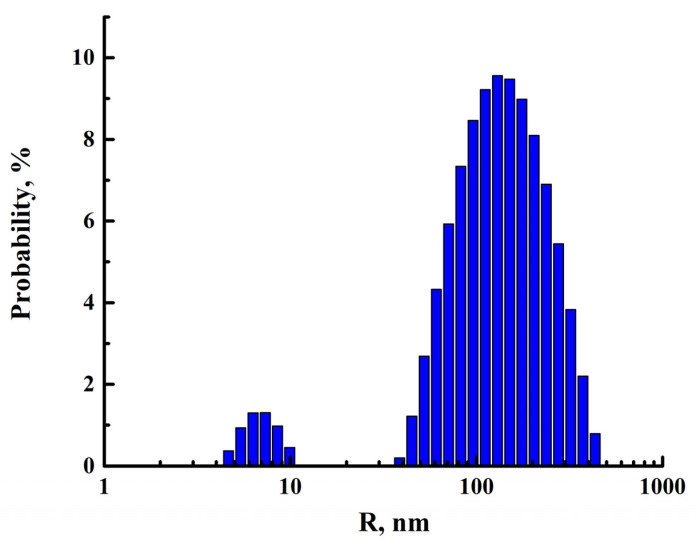
Size distribution of right-angle light scatter for a PEBA (0.04 wt% in 1-butanol).

The facility of Photocor Complex (Photocor, Moscow, Russia) allows for the determination of the particle size in the range of 0.5 nm to 6 μm by the method of dynamic light scattering and the molecular weight of polymers in the range of 10^3^–10^12^ g/mol by the analysis of static light scattering. The study of static and dynamic light scattering was carried out under the determined conditions (presented in more detail in Section 2.4): wavelength of the radiation source of 445 nm, temperature of 25 °C, scattering angles from 40 to 140° in increments of 10°, and polymer concentration of 0.0994, 0.0796, 0.0598 and 0.0397 wt%. A particle size distribution plot calculated from experimental dynamic light scattering data are shown in Figure 4. The bead size distribution of PEBA can be defined as nearly monomodal. Therefore, the exact average molecular weight cannot be determined. Experimental data on the study of dynamic and static light scattering of PEBA solution in 1-butanol are presented in Figures S10 and S11 and Tables S2–S8 of Supplementary Materials.

**Figure 5:**
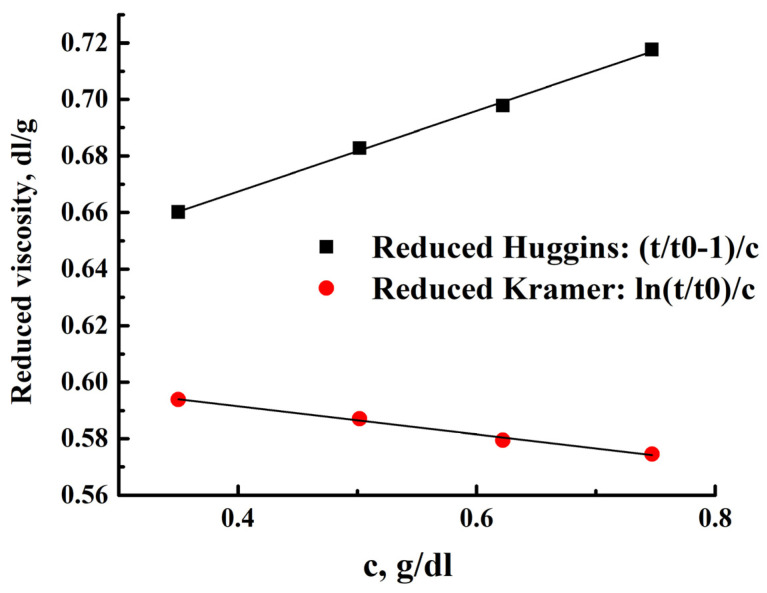
Reduced Huggins (black dots) and Kramer (red dots) viscosity versus concentration curve at 25 °C.

The Hoeppler principle was used to determine intrinsic viscosity and its dependence on temperature and concentration. This is the principle of a rolling ball in a closed capillary filled with a liquid sample. A viscometer measures the time taken for a solid ball to travel a certain distance through a tube placed at various angles to the horizontal. The values of reduced viscosity according to Huggins and Kramer (Figure 5) were calculated from the times of movement of the golden ball in solutions (Tables S9–S14 of Supplementary Materials) of different concentrations of the polymer at different temperatures.

For a polymer sample, the average molecular weight can be estimated from the viscosity and diffusion values for similar polymers with flexible chains. In the case of values A_0_ = 3.6 × 10^−10^, the molar mass of the polymer is M_Dη_ = 12,500 Da. Figure 5 shows the dependence of viscosity on polymer concentration for standard temperature (25 °C); the values of viscosity and Huggins’ and Kramer’s constants at different temperatures are presented in Table 2 and Table 3, respectively. The Huggins and Kramer viscosity dependencies converge to close values of the intrinsic viscosity. A decrease in the viscosity of solutions with increasing temperature, determined from the obtained dependencies, made it possible to prepare composite film materials with minimal defects.

**Figure 6:**
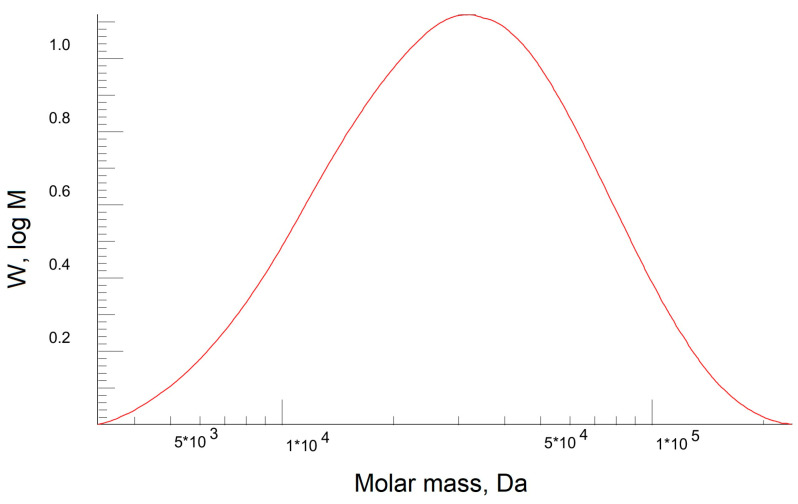
The molecular weight distribution of the PEBA polymer.

The molar mass and dispersion of the PEBA polymer were determined using gel permeation chromatography (GPH). Figure 6 shows the molecular weight distribution of the PEBA polymer.

It was found that the dispersion of the polymer was 1.8, the number average molecular weight (Mn) was 22 kDa, and the average molecular weight (Mw) was 39 kDa. This composition of the elastomer Pebax-2533 has previously shown the promise of creating films for membrane processes [106,107,108], but the calculated average molecular weight values with the viscosity values are also necessary to determine the Mark–Kun–Hauwink–Sakurada coefficients for further PEBA polymer studies with other molecular weight.

### 3.3. PEBA and PEBA/Ho-MOFs Membranes Investigation

The amount of MOFs introduced into the PEBA matrix was varied. Based on the literature, it was demonstrated that 2 wt% of MOFs in the PEBA matrix is sufficient for significant changes in membrane properties [87,107,109,110]. To investigate the effect of the synthesized Ho-MOFs on the MMMs properties, 2 wt% of Ho-MOFs were introduced into the PEBA matrix. FTIR, SEM, AFM, TGA and water contact angle measurements were used to characterize the obtained membranes. The ability of the membranes to retain dyes (Congo Red, Fuchsin, Glycine thymol blue, Methylene blue, Eriochrome Black T) was also investigated.

**Figure 7:**
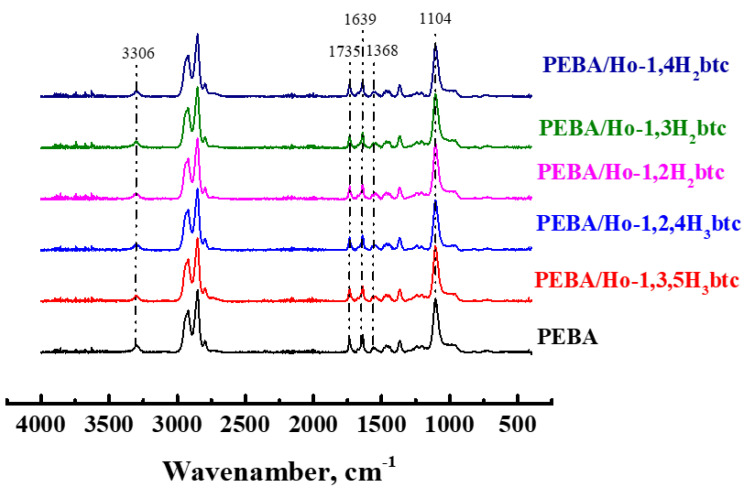
FTIR spectra of PEBA and PEBA/Ho-MOFs membranes.

The structural characteristics of PEBA and PEBA/Ho-MOFs membranes were studied by FTIR spectroscopy (Figure 7).

For pure PEBA membranes, the peaks at 3296 cm^−1^, 1735 cm^−1^, 1639 cm^−1^ and 1370 cm^−1^ are attributed to the stretching vibration of N–H, C=O, H–N–C=O, and C–N groups in polyamide segment, respectively [109,111]. The peak at 1104 cm^−1^ is assigned to the stretching vibration of the C–O–C group in the polyether segment [83,109,111,112]. For modified membranes, there were no new bands or band shifts in the FTIR spectra. Thus, the Ho-MOFs were physically blended with the PEBA matrix without chemical bonding. Such an interaction was also previously noted in works devoted to the development of PEBA/MOF membranes [111,112].

**Figure 8:**
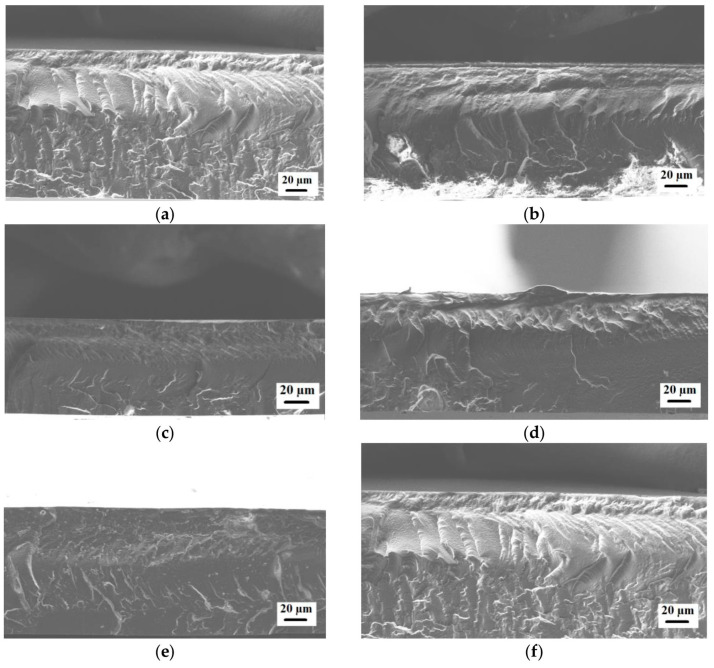
The cross-sectional SEM micrographs of membranes: (**a**) PEBA; (**b**) PEBA/Ho-1,3,5-H_3_btc; (**c**) PEBA/Ho-1,2,4-H_3_btc; (**d**) PEBA/Ho-1,2-H_2_bdc; (**e**) PEBA/Ho-1,3-H_2_bdc; (**f**) PEBA/Ho-1,4-H_2_bdc.

The inner structures of the PEBA and PEBA/Ho-MOFs membranes were studied by scanning electron microscopy (SEM). The cross-sectional SEM micrographs for the membranes are presented in Figure 8.

The presented SEM micrographs demonstrate the rough and ribbed structure of the cross-section for the unmodified PEBA membrane. Ho-MOF particles are not visible on the cross-section of modified membranes. The introduction of Ho-MOFs does not lead to defects in the polymer membranes. Also, Figure S12 of Supplementary Materials shows optical micrographs of the membrane surface obtained using a light microscope. Membranes containing 2 wt% Ho-MOF (Ho-1,3,5-H_3_btc and Ho-1,2-H_2_bdc) have the most uniform and visible distribution of Ho-MOF particles on the surface compared to others.

**Figure 9:**
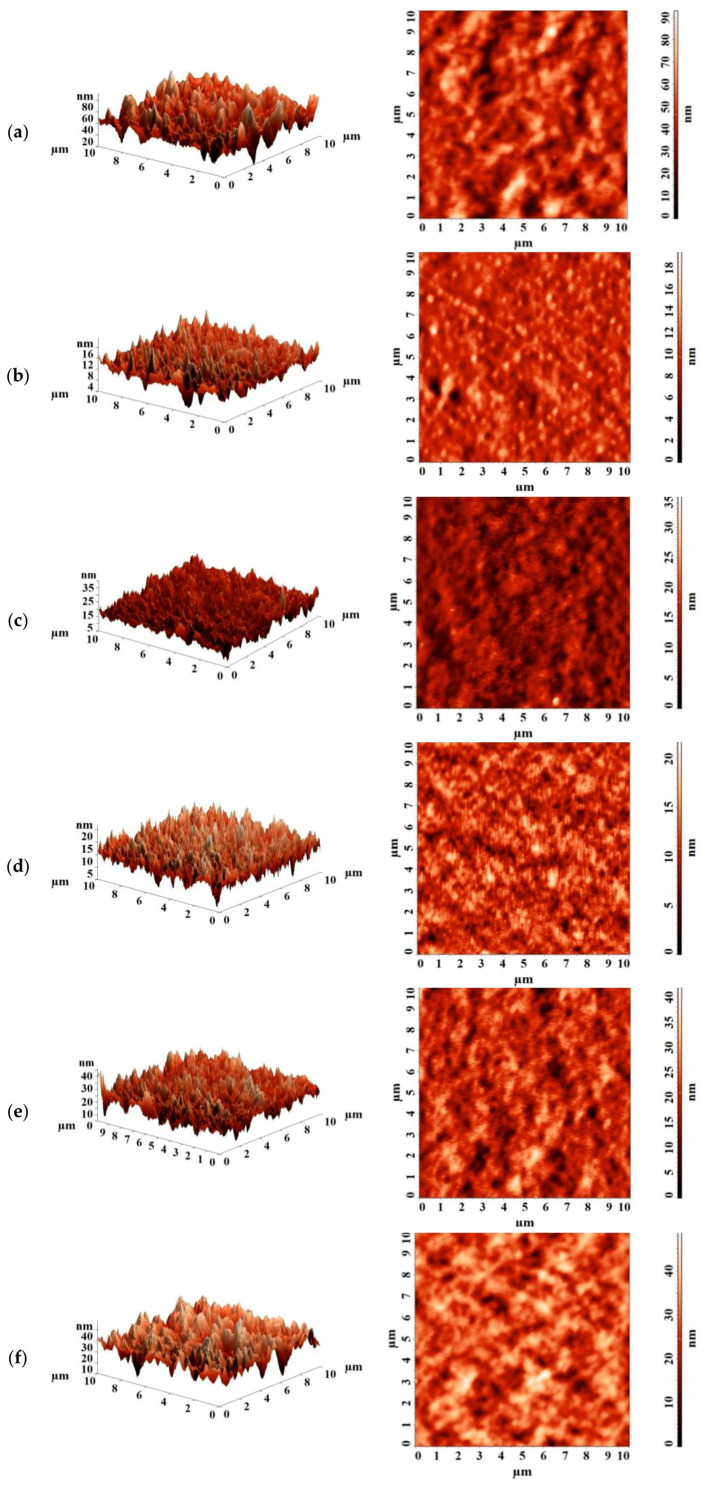
AFM images of membranes: (**a**) PEBA; (**b**) PEBA/Ho-1,3,5-H_3_btc; (**c**) PEBA/Ho-1,2,4-H_3_btc; (**d**) PEBA/Ho-1,2-H_2_bdc; (**e**) PEBA/Ho-1,3-H_2_bdc; (**f**) PEBA/Ho-1,4-H_2_bdc.

The surface roughness of the PEBA and PEBA/Ho-MOFs membranes was studied by atomic force microscopy (AFM). AFM images with a scan size of 10 × 10 μm are presented in Figure 9.

The average (Ra) and root-mean-squared (Rq) roughness of the PEBA and PEBA/Ho-MOFs membranes were calculated based on the AFM images (Figure 9) and presented in Table 4. To study the hydrophilic–hydrophobic balance of the surface of the developed membranes, contact angles of water were measured (Table 4).

For the modified PEBA/Ho-MOFs membranes, a decrease in roughness was noted compared to the pristine PEBA membrane. For the unmodified PEBA membrane, a contact angle of water was noted at 78°, which is close to the literature data [106,111]. Upon the introduction of Ho-MOFs, an increase in contact angles of water values was observed. It may be associated with hydrophobic ligands of modifiers. The same trend was previously demonstrated in works [106,107,111].

**Figure 10:**
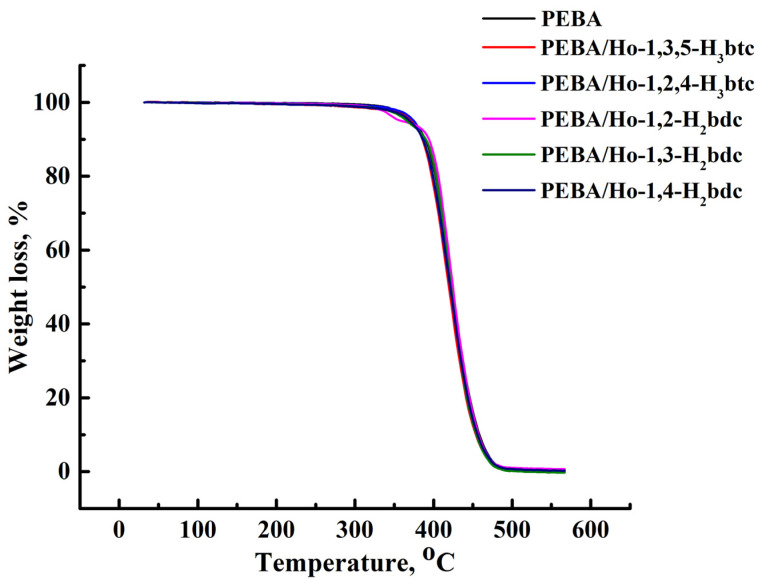
Thermogravimetric curves for PEBA and PEBA/Ho-MOFs membranes.

The thermal stability of the developed PEBA and PEBA/Ho-MOFs membranes was studied by TGA. The resulting thermograms are presented in Figure 10.

Each TGA curve shows a clear weight loss step. The decomposition temperature of the PEBA-based membrane was 360 °C, which is close to the published data [107,111]. The weight loss rate of the PEBA/Ho-MOFs mixed matrix membranes is almost the same as that of a pristine PEBA membrane because the addition of Ho-MOFs content is small. The obtained high thermal stability of all membranes allows us to use them in membrane processes at elevated temperatures.

**Figure 11:**
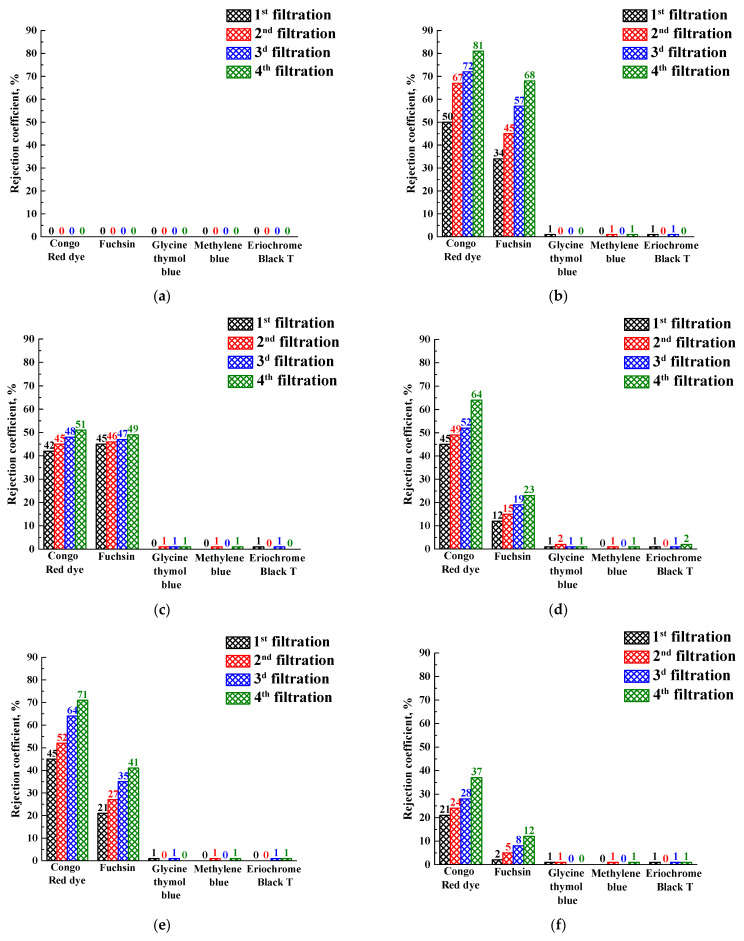
Rejection coefficients of dyes (Congo Red, Fuchsin, Glycine thymol blue, Methylene blue, Eriochrome Black T) in a 4-stage vacuum filtration for the membranes: (**a**) PEBA; (**b**) PEBA/Ho-1,3,5-H_3_btc; (**c**) PEBA/Ho-1,2,4-H_3_btc; (**d**) PEBA/Ho-1,2-H_2_bdc; (**e**) PEBA/Ho-1,3-H_2_bdc; (**f**) PEBA/Ho-1,4-H_2_bdc.

**Figure 12:**
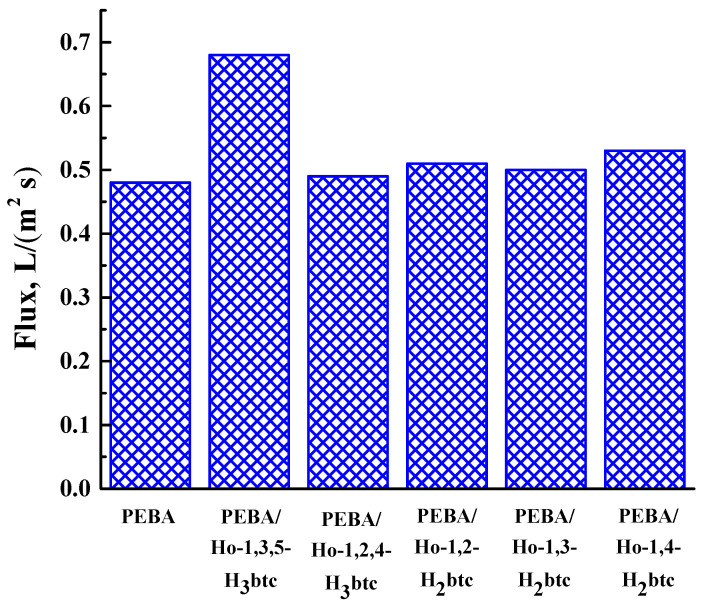
Fluxes of dilute dye solutions (Congo Red, Fuchsin, Glycine thymol blue, Methylene blue, Eriochrome Black T) in a four-stage vacuum filtration for PEBA and PEBA/Ho-MOF membranes.

To investigate the filtration properties, PEBA and PEBA/Ho-MOFs membranes were used as “filters” in vacuum filtration for the removal of dyes (Congo Red, Fuchsin, Glycine thymol blue, Methylene blue, Eriochrome Black T) from aqueous solution. The rejection coefficients and fluxes for membranes in a 4-stage vacuum filtration are shown in Figure 11 and Figure 12, respectively.

It was demonstrated that the pristine PEBA membrane did not reject dyes at all (0% rejection coefficient), i.e., the dye solutions passed through the membrane without any change in the concentration (Figure 11a). The rejection coefficient values of the Congo red for modified PEBA/Ho-MOF membranes were above 21% and raised with the increase of filtration stages. The rejection coefficients of Fuchsin for modified membranes were lower compared to the ones of the Congo Red but had the same increasing dependence during filtration. However, other dyes (Glycine thymol blue, Methylene blue, Eriochrome Black T) were not rejected by PEBA/Ho-MOF membranes. Membrane rejection may depend not only on the size of dye molecules (molecular weights of dyes are presented in Table S1 of Supplementary Materials) but also on the presence of specific functional groups in its structure. The rejection of Congo Red and Fuchsin by modified membranes is conditioned by non-ionized amino groups in their structures, which do not contain other dyes (Glycine thymol blue, Methylene blue, Eriochrome Black T). In addition, it should be noted that Congo Red and Fuchsin belong to different types of dyes: Congo Red is in the anionic form in an aqueous solution, and Fuchsin is in the cationic form. Thus, the retention ability of PEBA/Ho-MOF membranes for dyes with amino groups may be explained by the presence of vacancies in Ho-MOF structures, resulting in an increased number of uncoordinated carboxyl groups of linkers. The presence of such vacancies may be due to synthesis in ethanol medium coordinated to the holmium node [113,114,115,116]. The other studied dyes (Glycine thymol blue, Methylene blue, Eriochrome Black T) also have different natures (cationic, anionic or neutral) but do not contain amino groups that let dyes penetrate without their retention.

During all filtration cycles of dilute dye solutions (Congo Red, Fuchsin, Glycine thymol blue, Methylene blue, Eriochrome Black T), each membrane demonstrated the fluxes for every stage close in values. Thus, the averaged flux values are presented in Figure 12.

The introduction of Ho-MOFs into PEBA led to the flux increase compared with the pristine PEBA membrane (Figure 12). It could be due to the porous structure of the Ho-MOFs. The highest rejection coefficients of Congo Red and Fuchsin and flux were observed for the MMM containing the Ho-1,3,5-H_3_btc, which could be attributed to the uniform distribution of the Ho-1,3,5-H_3_btc particles in the PEBA matrix because of its structural peculiarity.

Thus, it was shown that the introduction of Ho-MOFs into the PEBA matrix could be a perspective for the development of MMMs for the effective removal of dyes. As a continuation of this work, membranes based on PEBA modified with Ho-MOFs will be investigated in membrane processes such as pervaporation and nanofiltration.

## 4. Conclusions

In this study, new metal-organic frameworks (MOFs) based on benzenecarboxylic acids as linkers for the holmium node were synthesized. The solvothermal synthesis in an atypical solvent (ethanol) of a series of Ho-MOFs with linkers—different benzoic acids allowed to obtain Ho-MOFs with atypical structures investigated by XRD, SEM, BET and IR spectroscopy methods. Two Ho-MOFs (Ho-1,2,4-H_3_btc and Ho-1,2-H_2_bdc) had amorphous X-ray structures, and Ho-1,3,5-H_3_btc, Ho-1,3-H_2_bdc and Ho-1,4-H_2_bdc with crystalline X-ray structures were obtained. It was demonstrated that all synthesized Ho-MOFs were microporous materials with low specific surface area, except for Ho-1,2-H_2_bdc, which was found to have a high specific surface area. This was mainly due to the synthesis method of the obtained materials, namely, the synthesis in ethanol.

The synthesized Ho-MOFs were evaluated as modifiers for a commercial polymer polyether block amide Pebax-2533. The PEBA properties were investigated by dynamic and kinematic viscosity, static and dynamic light scattering and gel permeation chromatography methods. Polymer particles in the 1-butanol solution had an equivalent spherical size of approximately 100 nm. The molecular weight distribution of the polymer chains was close to monomodal, as the size distribution of the particles was quite narrow (also confirmed by chromatography). The dynamic behavior of the solution and the dependence on static and dynamic viscosity of PEBA solutions confirmed that the polymer belonged to the class of flexible chains. The Kramer and Higgins coefficients were determined for these solutions over a fairly wide range of temperatures and concentrations.

The mixed matrix dense membranes based on PEBA/Ho-MOFs composites were developed and characterized by FTIR spectroscopy, scanning electron and atomic force microscopy, thermogravimetric analysis and water contact angle measurements. It was obtained that the introduction of Ho-MOFs into the PEBA matrix led to a decrease in surface roughness and an increase in hydrophobicity, which could be related to the hydrophobic ligands of the modifiers. High thermal stability was observed for all developed membranes, allowing their use in membrane processes at elevated temperatures. The introduction of Ho-MOFs into the PEBA caused a change in the transport properties in four-stage vacuum filtration of dye aqueous solutions (Congo Red, Fuchsin, Glycine thymol blue, Methylene blue, Eriochrome Black T). For all modified membranes, the increase of the flux was noted, but the rise of rejection coefficients was observed only for dyes containing amino groups (Congo Red and Fuchsin). It could be explained by the fact that non-ionized amino groups in dye structures coordinated carboxyl groups of linkers in Ho-MOFs. The optimal Ho-MOF modifier for PEBA was Ho-1,3,5-H_3_btc. This membrane demonstrated the highest rejection coefficients (81% Congo Red and 68% Fuchsin) and flux (0.7 L/(m^2^s)) due to Ho-1,3,5-H_3_btc needle-shaped structure, crystal morphology and uniform distribution of particles in the polymer matrix. Thus, it was demonstrated that synthesized novel Ho-MOFs in ethanol are perspective modifiers for PEBA membranes, in particular, for dye filtration.

## Figures and Tables

**Table 1:** Specific surface area of the substances obtained.

Ho-MOF	Specific Surface Area, m^2^/g
Ho-1,3,5-H_3_btc	10
Ho-1,2,4-H_3_btc	1
Ho-1,2-H_2_bdc	241
Ho-1,3-H_2_bdc	10
Ho-1,4-H_2_bdc	1

**Table 2:** Intrinsic viscosities.

T, °C	[η], dL/g		
	Huggins	Kramer	Average
10	0.615	0.617	0.616
20	0.611	0.613	0.612
25	0.610	0.612	0.611
30	0.610	0.610	0.610
40	0.606	0.607	0.607
50	0.604	0.603	0.604

**Table 3:** Huggins’ and Kramer’s constants.

T, °C	Huggins	Kramer
10	0.411	−0.119
20	0.392	−0.129
25	0.384	−0.134
30	0.365	−0.143
40	0.362	−0.145
50	0.346	−0.153

**Table 4:** The surface roughness characteristics and contact angle of water for PEBA and PEBA/Ho-MOFs membranes.

Membrane	Ra, nm	Rq, nm	Contact Angle of Water, °
PEBA	9.4 ± 0.5	11.9 ± 0.6	78 ± 1
PEBA/Ho-1,3,5-H_3_btc	1.3 ± 0.1	1.7 ± 0.1	85 ± 1
PEBA/Ho-1,2,4-H_3_btc	2.4 ± 0.1	3.0 ± 0.1	86 ± 1
PEBA/Ho-1,2-H_2_bdc	2.0 ± 0.1	2.6 ± 0.1	86 ± 1
PEBA/Ho-1,3-H_2_bdc	3.9 ± 0.2	5.0 ± 0.2	87 ± 1
PEBA/Ho-1,4-H_2_bdc	4.8 ± 0.2	6.1 ± 0.2	86 ± 1

## Data Availability

The data presented in this study are available on request from the corresponding author.

## Supplementary Materials

The following supporting information can be downloaded at: https://www.mdpi.com/article/10.3390/polym15183834/s1, Figure S1: Structural formula of (**a**) 1,3,5-H_3_btc; (**b**) 1,2,4-H_3_btc; (**c**) 1,2-H_2_bdc; (**d**) 1,3-H_2_bdc; (**e**) 1,4-H_2_bdc; Figure S2. Shifted powder diffraction patterns for (**a**) Ho-1,4-H_2_bdc (black line, synthesized in this work) and Y-1,4-H_2_bdc (blue line, simulated from cif-file [95]); (**b**) Ho-1,3-H_2_bdc (black line, synthesized in this work) and Al-1,3-H_2_bdc (blue line, simulated from cif-file [96]).; (**c**) Ho-1,3,5-H_3_btc (black line, synthesized in this work) and Ho-1,3,5-H_3_btc (blue line, simulated from cif-file [97]); Figure S3. Structure of (**a**) Ho-1,4-H_2_bdc simulated from *cif*-file [95], (**b**) Ho-1,3-H_2_bdc simulated from *cif*-file [96], (**c**) Ho-1,3,5-H_3_btc simulated from *cif*-file [96]; Figure S4. EDX spectra for SEM micrographs (presented in Figure 2) of Ho-1,3,5-H3btc MOF; Figure S5. EDX spectra for SEM micrographs (presented in Figure 2) of Ho-1,2,4-H3btc MOF; Figure S6. EDX spectra for SEM micrographs (presented in Figure 2) of Ho-1,2-H2bdc MOF; Figure S7. EDX spectra for SEM micrographs (presented in Figure 2) of Ho-1,3-H2bdc MOF; Figure S8. EDX spectra for SEM micrographs (presented in Figure 2) of Ho-1,4-H_2_bdc MOF; Figure S9. Nitrogen adsorption–desorption isotherm of Ho-MOF: (**a**) Ho-1,3,5-H_3_btc; (**b**) Ho-1,2,4-H_3_btc; (**c**) Ho-1,2-H_2_btc; (**d**) Ho-1,3-H_2_btc; (**e**) Ho-1,4-H_2_btc; Figure S10: Plots (**a**) of the reciprocal relaxation times of the particle concentration fluctuations (τ_1_) versus the square of the wave vector and (**b**) the dependence of the translational diffusion coefficients (D_t1_) on the concentration for the fast mode; Figure S11: (**a**) Plot of reciprocal relaxation times of particle concentration fluctuations (τ_2_) versus the square of the wave vector and (**b**) plot of translational diffusion coefficients (D_t2_) versus concentration (right) for the slow mode; Figure S12: Optical micrographs for PEBA/Ho-MOFs membranes: (**a**) PEBA/Ho-1,3,5-H_3_btc; (**b**) PEBA/Ho-1,2,4-H_3_btc; (**c**) PEBA/Ho-1,2-H_2_bdc; (**d**) PEBA/Ho-1,3-H_2_bdc; (**e**) PEBA/Ho-1,4-H_2_bdc; Table S1. Structural formula, molecular weight, maximum absorption wavelength of the dyes used; Table S2: Integrated light scattering intensity for the solvent (1-butanol) and the standard (toluene) (445 nm, 25 °C); Table S3: Measured values used for the calculation of the physical parameters of the solvent (1-butanol) and of the standard (toluene) at 25 °C; Table S4: Characteristic relaxation times of particle concentration fluctuations (τ1) in the scattered volume (fast mode); Table S5: Translation coefficient (Dt1) and radius of equivalent sphere (Rh1) corresponding to the fast mode; Table S6: Characteristic relaxation times of particle concentration fluctuations (τ_2_) in the scattered volume (slow mode); Table S7: Translational diffusion coefficient (D_t2_) and hydrodynamic radius of an equivalent sphere (R_h2_) corresponding to the slow mode; Table S8: Integrated intensity of the light scattering (I) for polymer solutions and its standard deviation (St.D. I); Table S9: Results of measurement of relative and reduced viscosity at 10 °C; Table S10: Results of measurement of relative and reduced viscosity at 20 °C; Table S11: Results of measurement of relative and reduced viscosity at 25 °C; Table S12: Results of measurement of relative and reduced viscosity at 30 °C; Table S13: Results of measurement of relative and reduced viscosity at 40 °C; Table S14: Results of measurement of relative and reduced viscosity at 50 °C.
