# Supplementary material for: Holmium-Containing Metal-Organic Frameworks as Modifiers for PEBA-Based Membranes
Source: Polymers (Basel). 2023 Sep 20;15(18):3834. doi: 10.3390/polym15183834 (PMC10534401; doi:10.3390/polym15183834)
Supplement: Supplementary file 1 [file polymers-15-03834-s001.zip › polymers-2559553-supplementary.pdf]

Supplementary Materials

Holmium-containing metal-organic frameworks as modifiers for PEBA-based membranes

Anna Kuzminova [1], Mariia Dmitrenko [1], Kirill Salomatin [1], Olga Vezo [1], Sergey Kirichenko [1], Semyon Egorov [1], Marina Bezrukova [2], Anna Karyakina [1], Alexey Eremin [2], Ekaterina Popova [2,3,4], Anastasia Penkova [1], and Artem Selyutin [1,*]

[1] Saint-Petersburg State University, 7/9 Universitetskaya Emb., Saint-Petersburg, 199034, Russia
[2] Institute of Macromolecular Compounds, Russian Academy of Sciences, 31 Bolshoy pr., St. Petersburg 199004, Russia
[3] Saint-Petersburg State Institute of Technology (Technical University), 24-26/49 letter A Moskovski ave., St. Petersburg 190013, Russia
[4] Saint-Petersburg State Chemical Pharmaceutical University, 14 Akad. Popova st., St. Petersburg 197022, Russia

* Correspondence: a.selyutin@spbu.ru ; selutin@inbox.ru

S1. Materials

Figure S1 shows the ligands that have been used to synthesize metal-organic frameworks based on holmium.

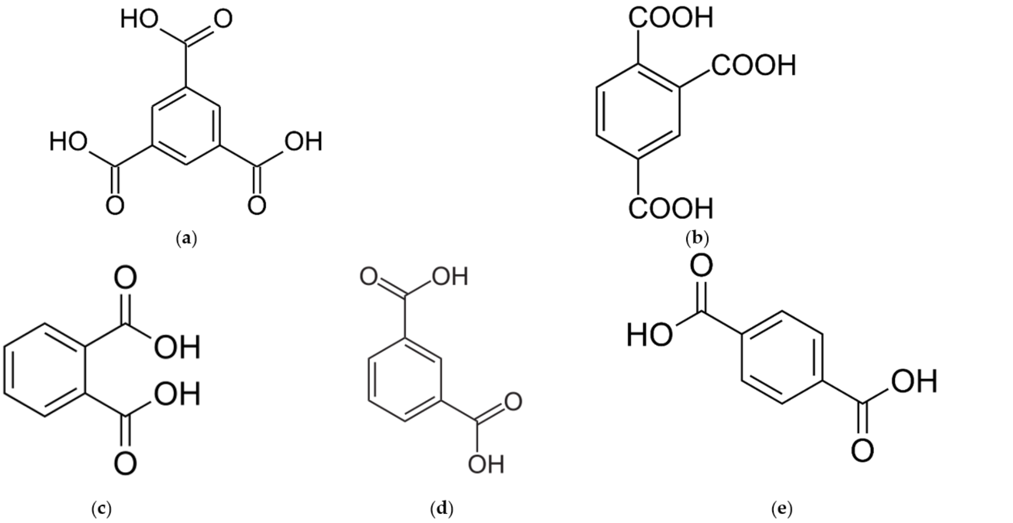

Figure S1. Structural formula of ligands (**a**) 1,3,5-H_3btc; (**b**) 1,2,4-H_3btc; (**c**) 1,2-H_2bdc; (**d**) 1,3-H_2bdc; (**e**) 1,4-H_2bdc.

Table S1 shows characteristics of dyes, such as structural formula, molecular weight, maximum absorption wavelength.

Table S1. Structural formula, molecular weight, maximum absorption wavelength of the dyes used.

Dye	Structural formula	Molecular weight, g/mol	Maximum absorption wavelength, nm
Congo Red dye	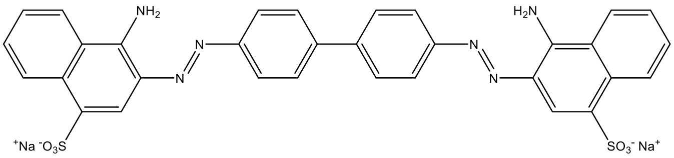	696.67	500
Fuchsin	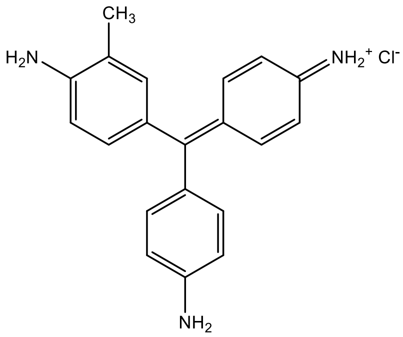	290.32	550
Glycine thymol blue	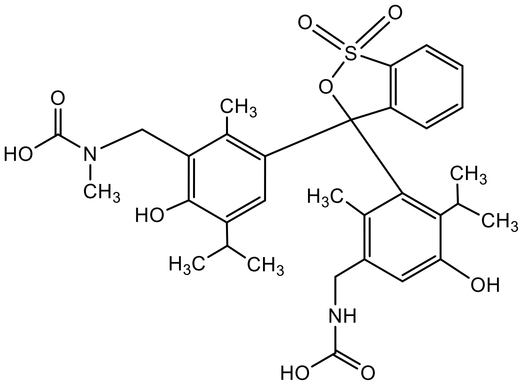	640.74	600
Methylene blue	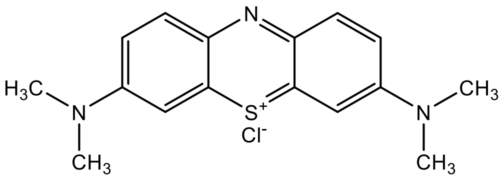	319.85	555
Eriochrome Black T	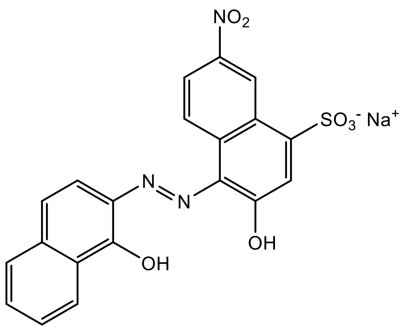	461.38	540

S2. Ho-MOFs Investigation

Shifted powder diffraction patterns for Ho-1,4-H_2bdc, Ho-1,3-H_2bdc, and Ho-1,3,5-H_3btc are presented in Figure S2.

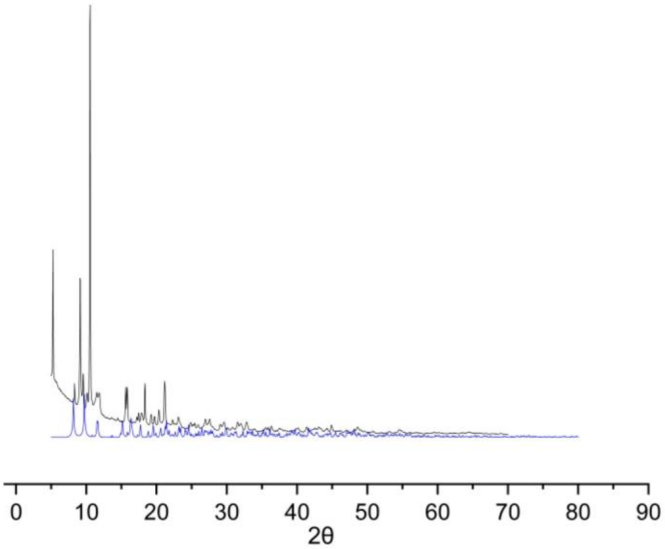

(**a**)

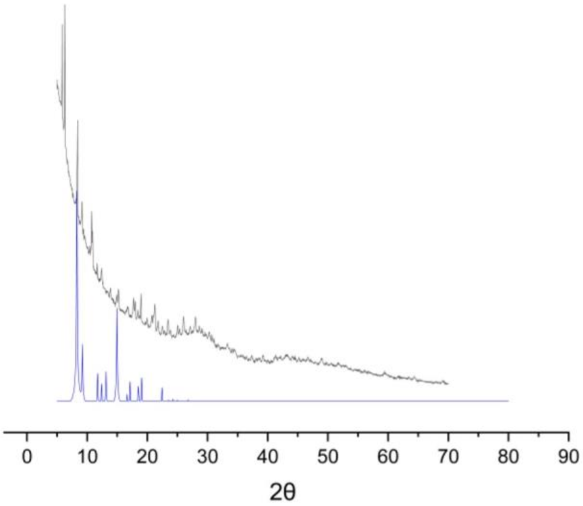

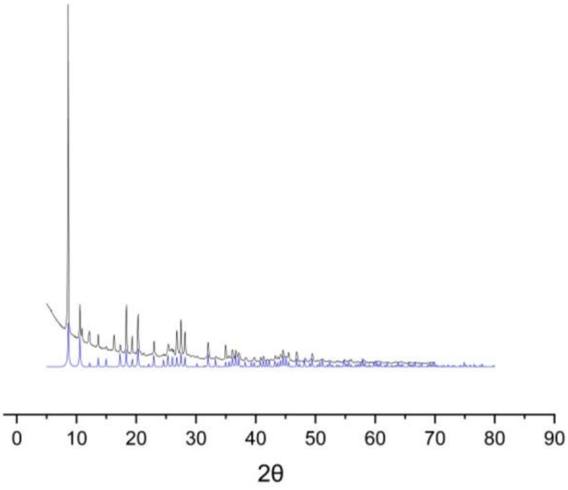

(**b**) (**c**)

Figure S2. Shifted powder diffraction patterns for (**a**) Ho-1,4-H_2bdc (black line, synthesized in this work) and Y-1,4-H_2bdc (blue line, simulated from *cif*-file [1]); (**b**) Ho-1,3-H_2bdc (black line, synthesized in this work) and Al-1,3-H_2bdc (blue line, simulated from *cif*-file [2]).; (**c**) Ho-1,3,5-H_3btc (black line, synthesized in this work) and Ho-1,3,5-H_3btc (blue line, simulated from *cif*-file [3]).

The structures of the synthesized MOFs are shown in Figure S3.

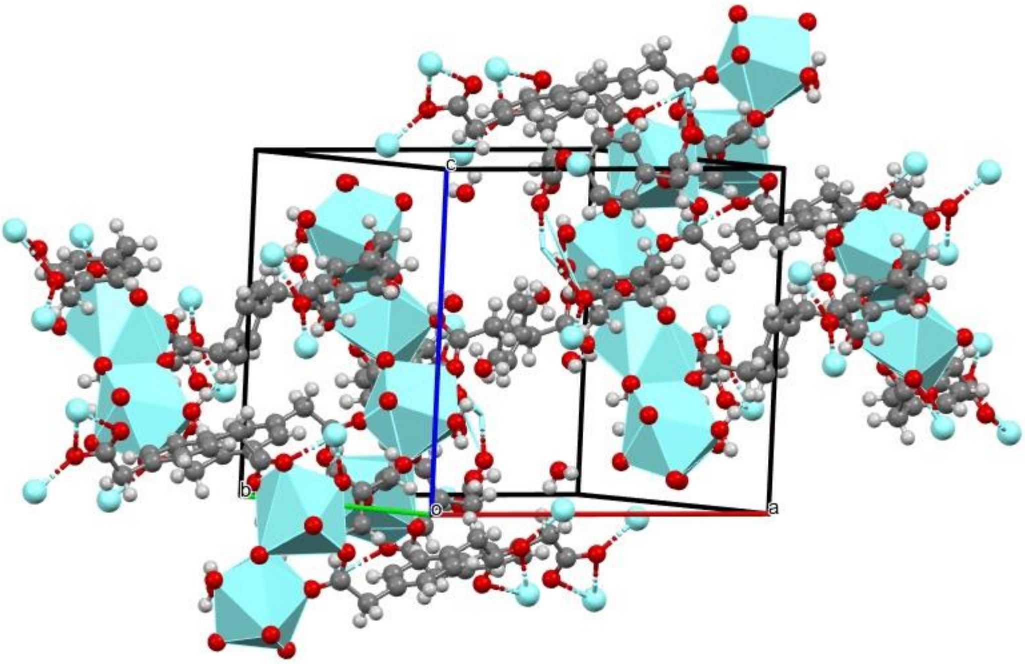

(**a**)

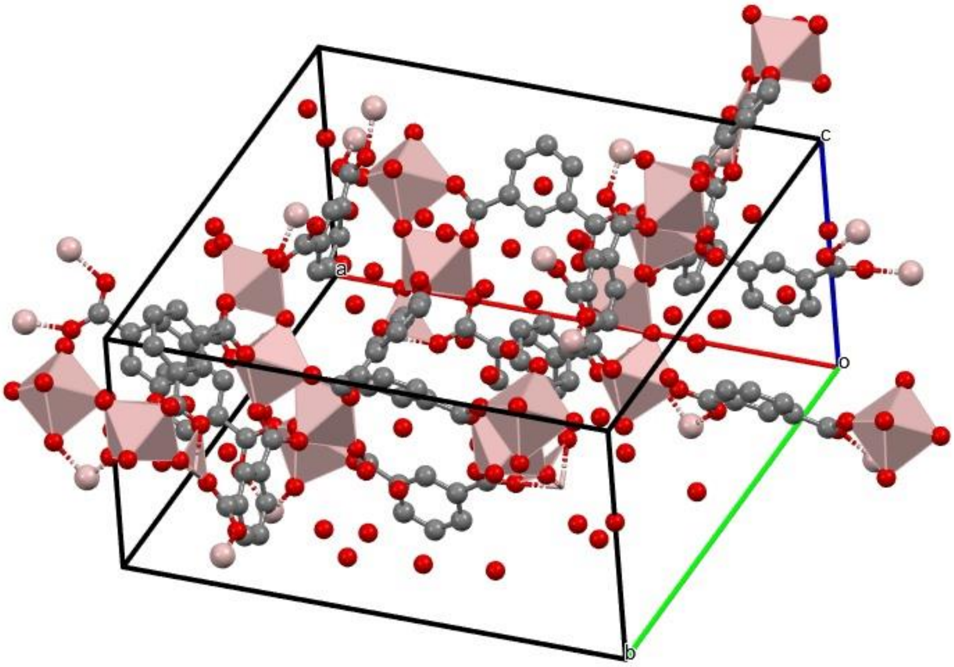

(**b**)

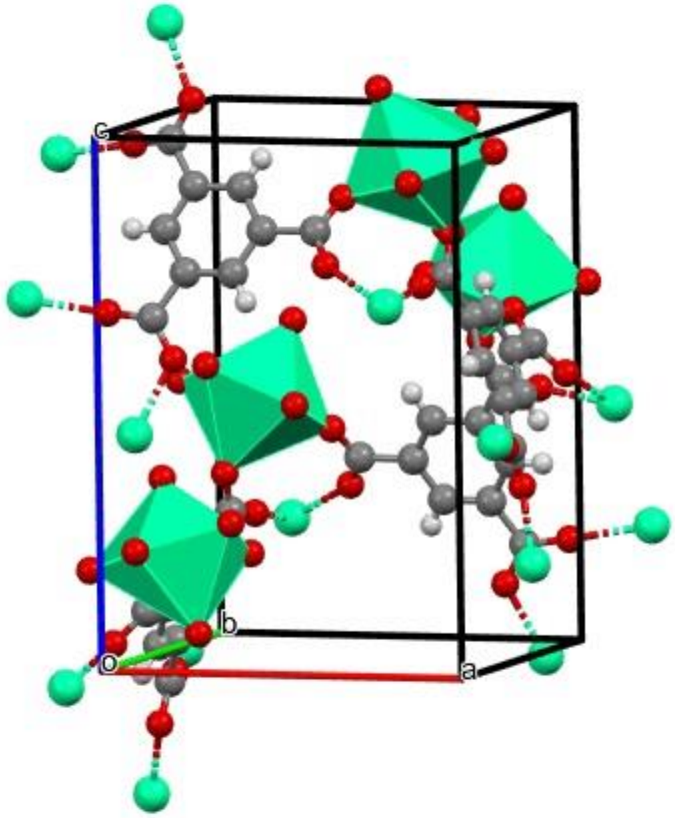

(**c**)

Figure S3. Structure of (**a**) Ho-1,4-H_2bdc simulated from *cif*-file [1], (**b**) Ho-1,3-H_2bdc simulated from *cif*-file [2], (**c**) Ho-1,3,5-H_3btc simulated from *cif*-file [2].

Scanning electron microscopy (SEM) with additional energy dispersive elemental analysis (EDX) was performed on the synthesized Ho-MOFs. Figure S4 shows the EDX spectra for SEM micrographs (presented in Figure 2) of Ho-1,3,5-H_3btc MOF. Figure S5 shows the EDX spectra for SEM micrographs (presented in Figure 2) of Ho-1,2,4-H_3btc MOF. Figure S6 shows the EDX spectra for SEM micrographs (presented in Figure 2) of Ho-1,2-H_2bdc MOF. Figure S7 shows the EDX spectra for SEM micrographs (presented in Figure 2) of Ho-1,3-H_2bdc MOF. Figure S8 shows the EDX spectra for SEM micrographs (presented in Figure 2) of Ho-1,4-H_2bdc MOF.

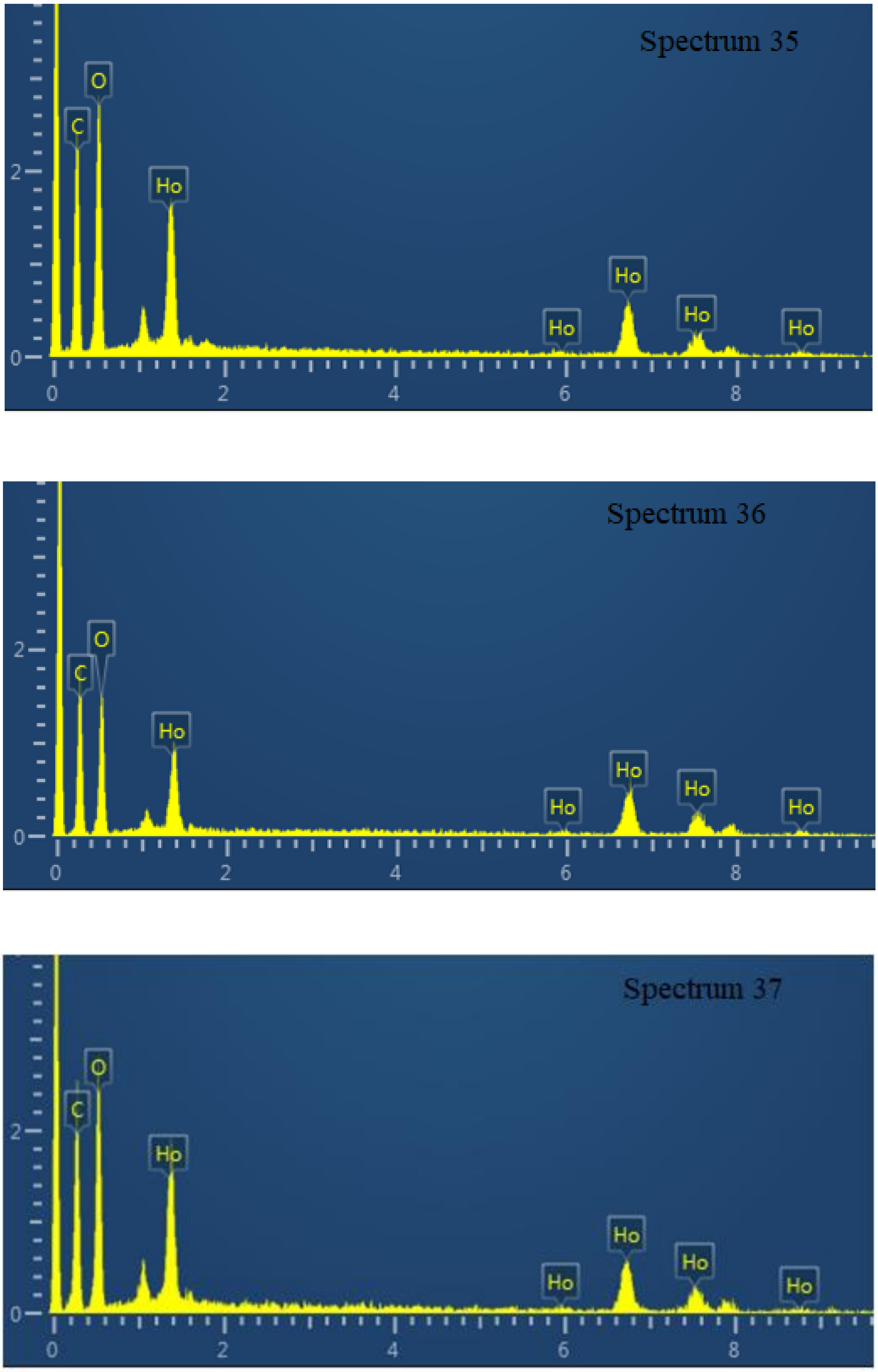

Figure S4. EDX spectra for SEM micrographs (presented in Figure 2) of Ho-1,3,5-H_3btc MOF.

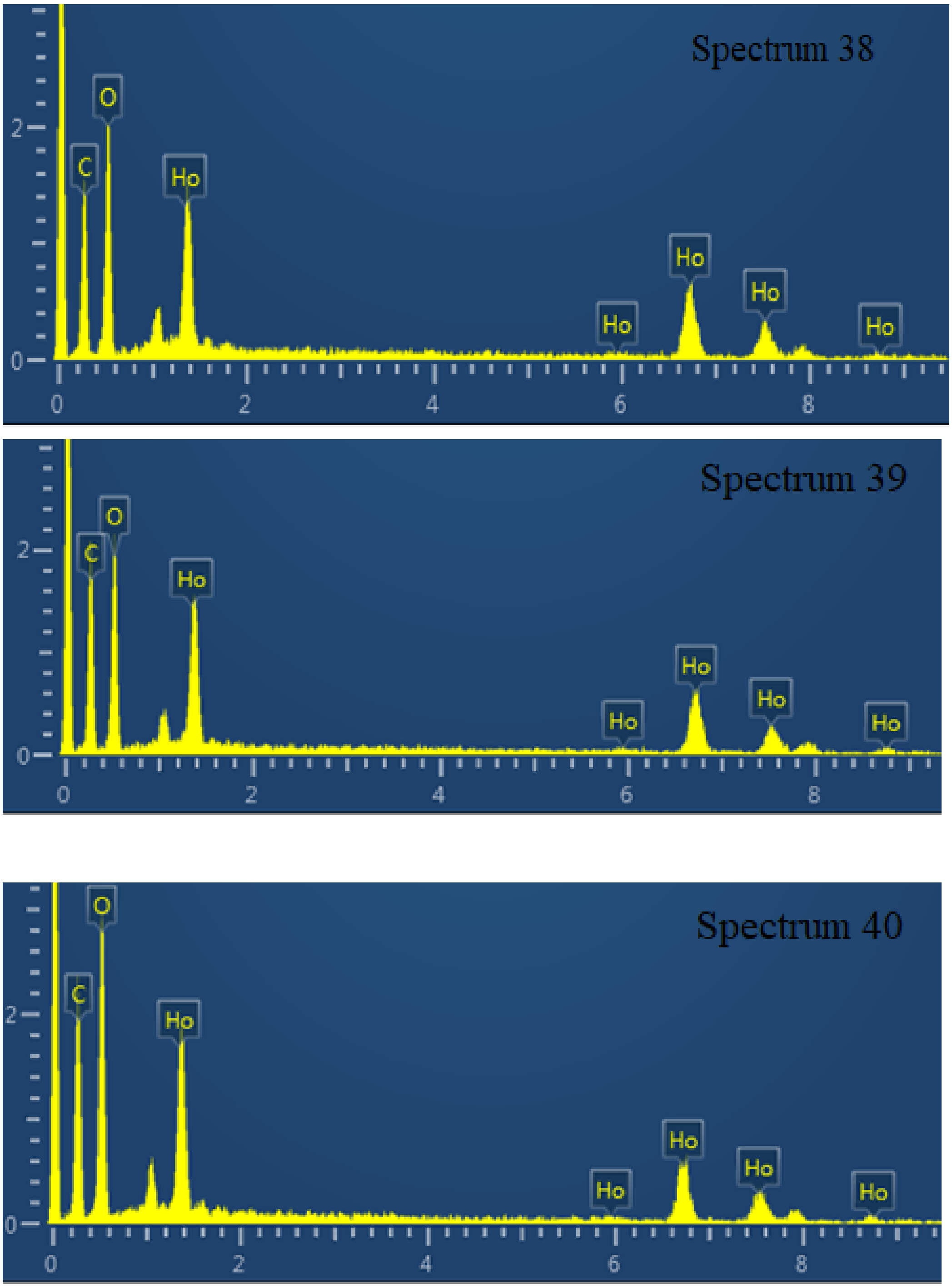

Figure S5. EDX spectra for SEM micrographs (presented in Figure 2) of Ho-1,2,4-H_3btc MOF.

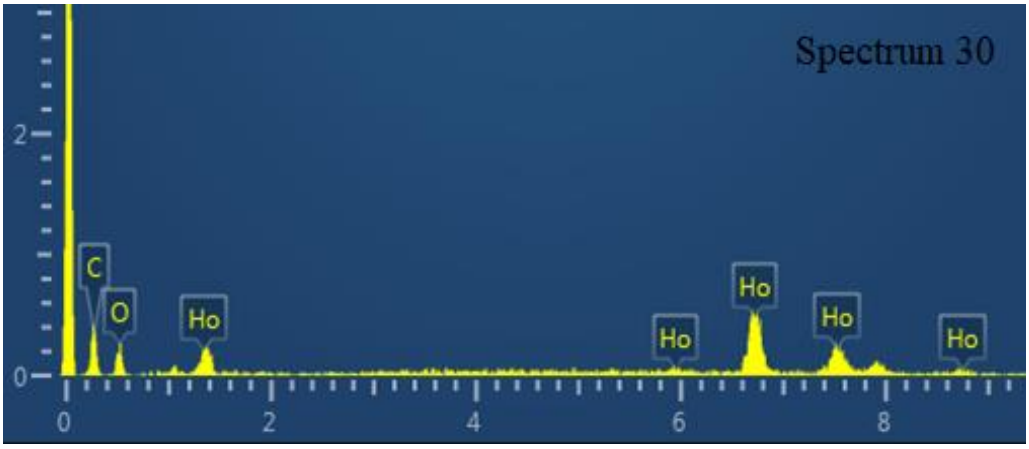
Spectrum 30
C
O
Ho
Ho
Ho
Ho
Ho
0
2
0
2
4
6
8

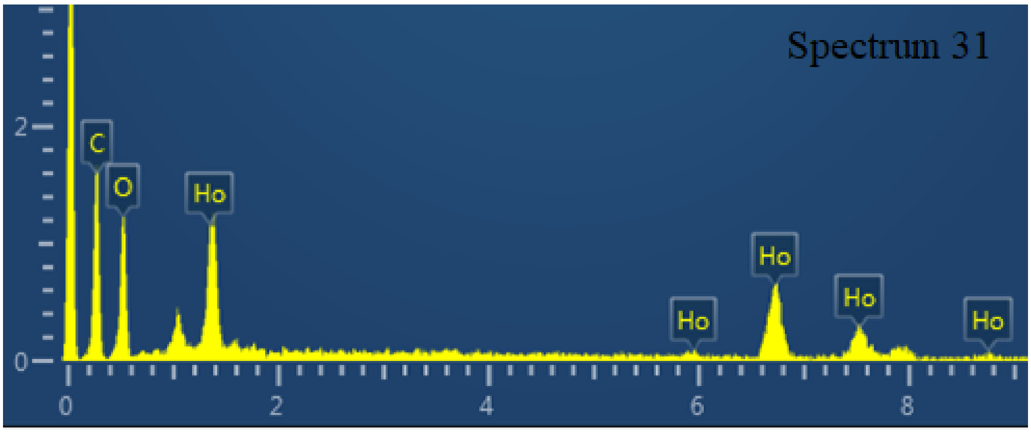
Spectrum 31
C
O
Ho
Ho
Ho
Ho
Ho
0
2
0
2
4
6
8

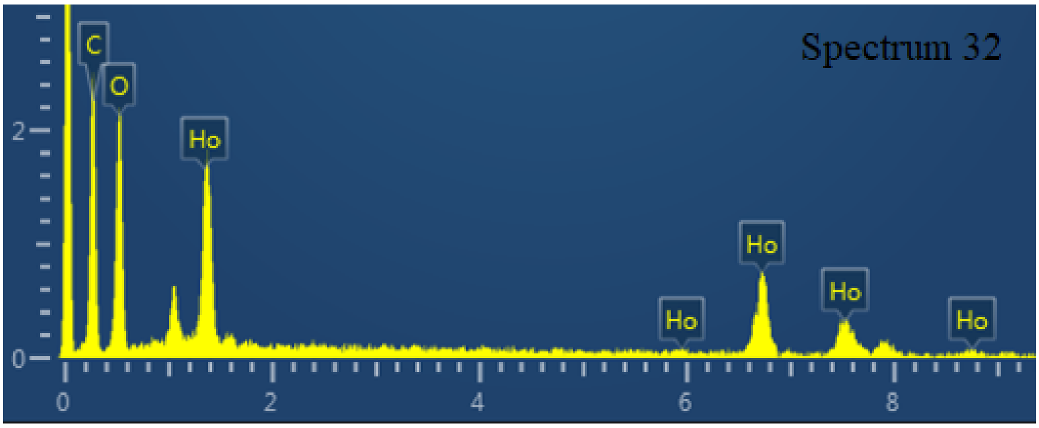
Spectrum 32
C
O
Ho
Ho
Ho
Ho
Ho
0
2
0
2
4
6
8

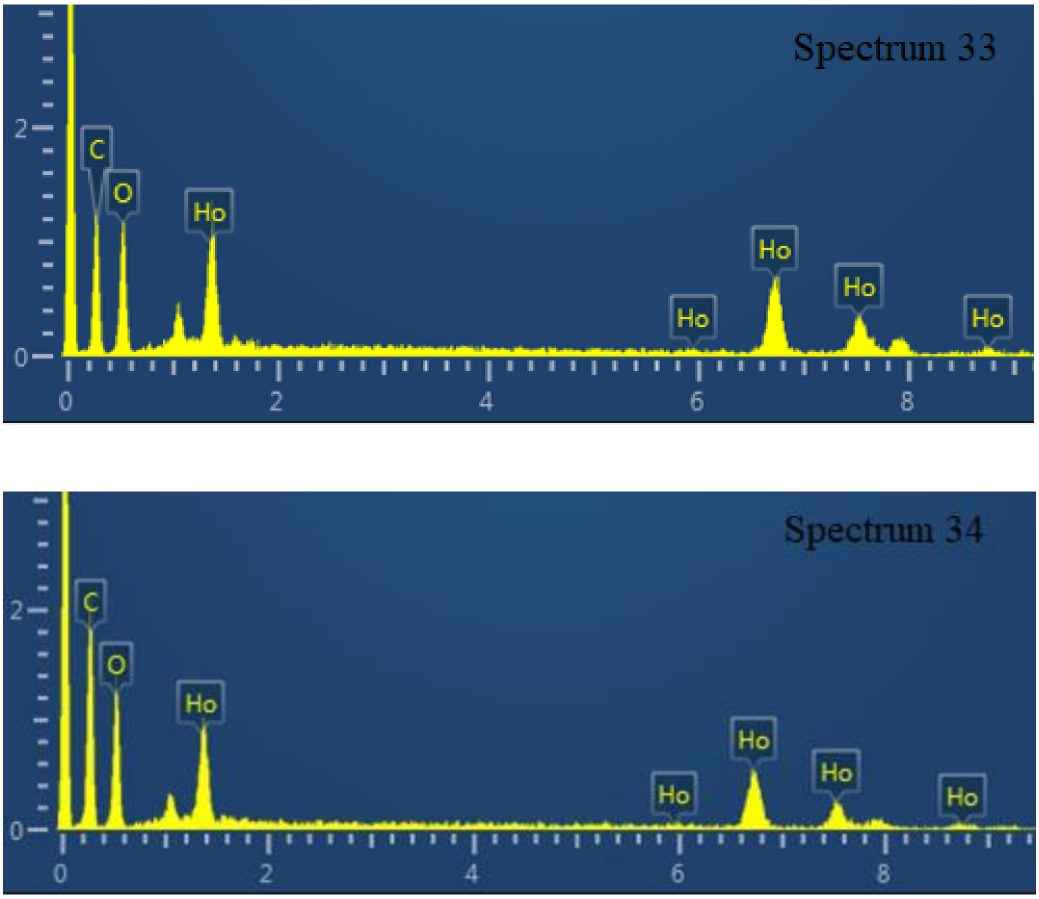

Figure S6. EDX spectra for SEM micrographs (presented in Figure 2) of Ho-1,2-H_2bdc MOF.

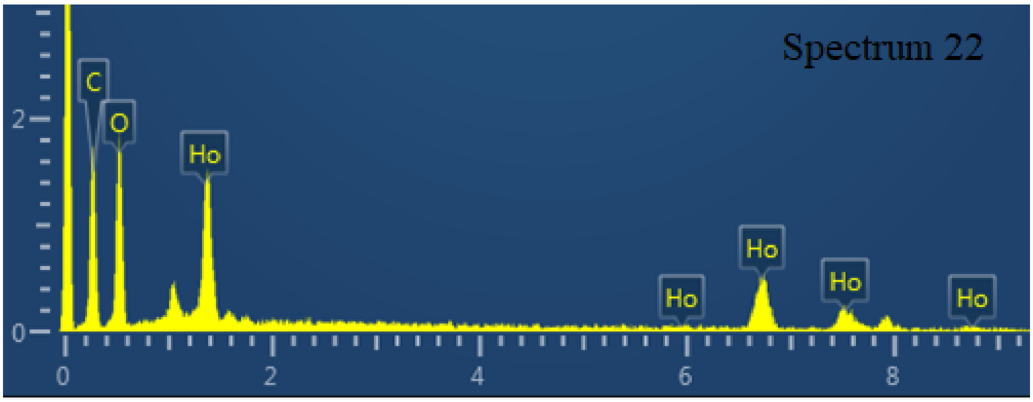

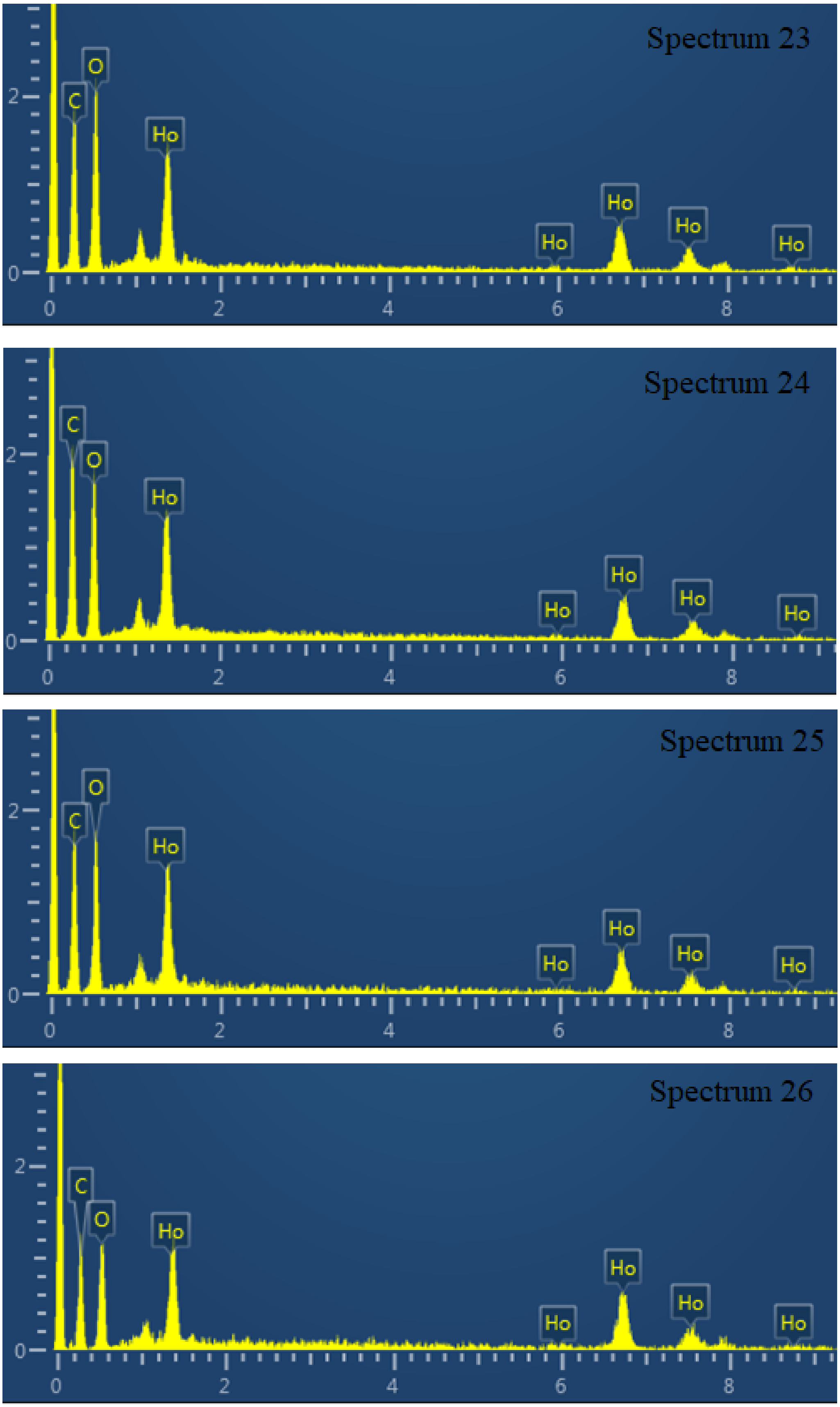

Figure S7. EDX spectra for SEM micrographs (presented in Figure 2) of Ho-1,3-H_2bdc MOF.

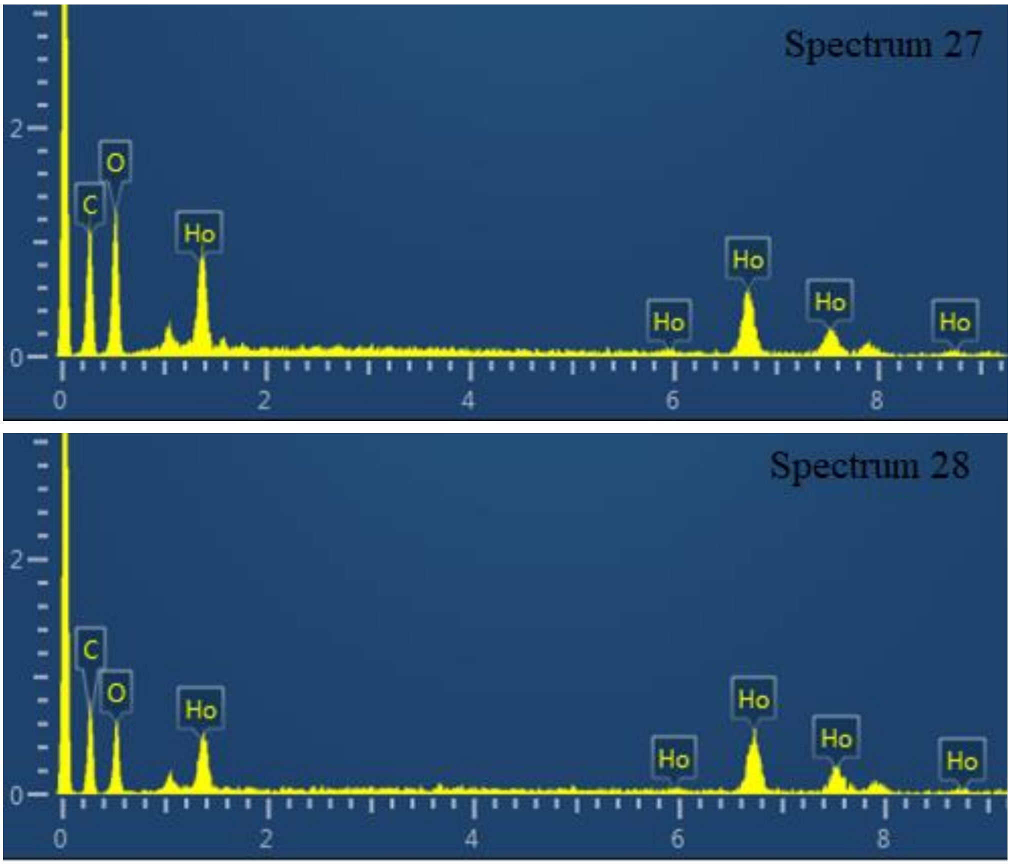

Figure S8. EDX spectra for SEM micrographs (presented in Figure 2) of Ho-1,4-H_2bdc MOF.

The Ho-MOFs were studied by low temperature nitrogen adsorption. Figure S9 shows the nitrogen adsorption-desorption isotherm of Ho-MOFs.

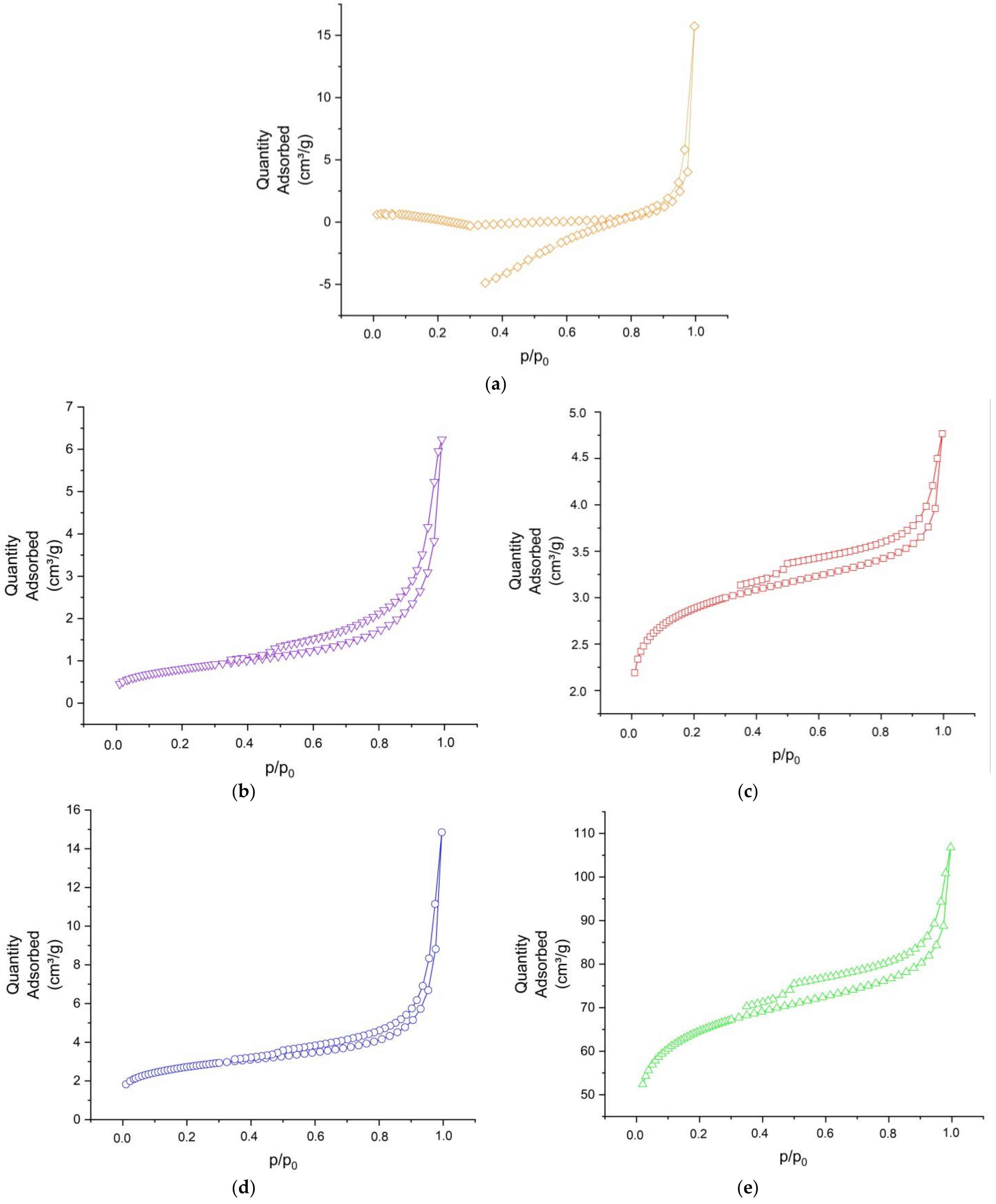

Figure S9. Nitrogen adsorption-desorption isotherm of Ho-MOF: (**a**) Ho-1,3,5-H_3btc; (**b**) Ho-1,2,4-H_3btc; (**c**) Ho-1,2-H_2btc; (**d**) Ho-1,3-H_2btc; (**e**) Ho-1,4-H_2btc.

S3. PEBA Investigation

The particle size of PEBA polymer was investigated by the method of dynamic light scattering. The molecular weight of PEBA polymer was studied by the analysis of static light scattering. Table S2 shows integrated light scattering intensity for the solvent (1-butanol) and the standard (toluene) at 445 nm and 25 °C.

Table S2. Integrated light scattering intensity for the solvent (1-butanol) and the standard (toluene) (445 nm, 25°C).

Θ, °	1-butanol		Toluene	
	I	St.D.I, %	I	St.D. I, %
40	32480	0.6	105111	0.4
50	24950	0.7	81820	0.5
60	20855	0.7	68881	0.4
70	18379	0.8	61046	0.6
80	17041	0.9	56302	0.6
90	16324	0.8	53932	0.5
100	16023	0.9	53152	0.5
110	16259	0.8	53796	0.4
120	17031	0.9	56423	0.5
130	18411	0.8	61047	0.4
140	20798	0.8	68893	0.4

Table S3 shows measured values used for the calculation of the physical parameters of the solvent (1-butanol) and of the standard (toluene) at 25 °C.

Table S3. Measured values used for the calculation of the physical parameters of the solvent (1-butanol) and of the standard (toluene) at 25 °C.

	ϱ, g/cm³	η, mPa·c	$n_{436,4\ nm}$	$n_{589,3\ nm}$	$n_{657,2\ nm}$
1-butanol	0.80842	2.9327	1.405484	1.397054	1.395059
Toluene	-		1.51459	1.49398	1.48950

Table S4 shows characteristic relaxation times of particle concentration fluctuations (τ_1) in the scattered volume (fast mode). Figure S10 shows plots of the reciprocal relaxation times of the particle concentration fluctuations (τ_1) versus the square of the wave vector and the dependence of the translational diffusion coefficients (D_{t1}) on the concentration for the fast mode. Table S5 shows translation coefficient (D_{t1}) and radius of equivalent sphere (R_{h1}) corresponding to the fast mode.

Table S4. Characteristic relaxation times of particle concentration fluctuations (τ1) in the scattered volume (fast mode).

	**C, %**	**0.0994**	**0.0796**	**0.0598**	**0.0397**
**Θ, °**	**q^2*10^{14}, 1/м²**		**τ_1, mc**		
40	1.843	-	0.6770	0.4777	0.6130
50	2.814	0.5643	0.4530	0.5430	0.4637
60	3.938	0.2470	0.1930	0.4350	0.3843
70	5.182	0.4013	0.1743	0.1743	0.3827
80	6.509	0.2143	0.2143	0.1520	0.1547
90	7.876	0.1497	0.2880	0.1155	0.2880
100	9.244	0.1610	0.1633	0.1020	-
110	10.57	0.0867	0.1345	0.1350	0.1160
120	11.81	0.1160	0.0770	0.0860	0.0900
130	12.94	0.1350	-	-	0.0860

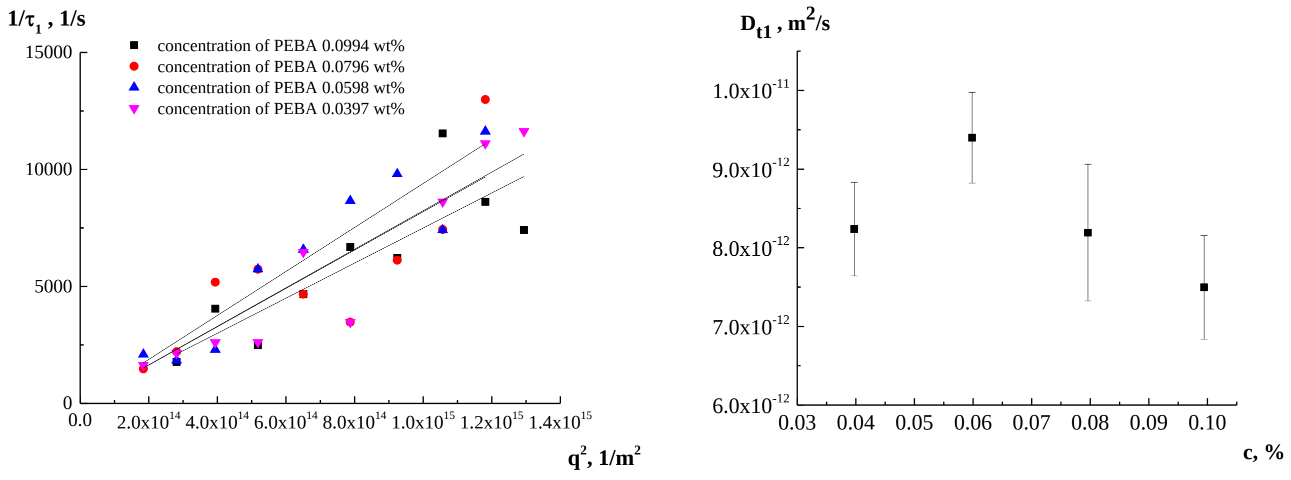

(**a**) (**b**)

Figure S10. Plots (**a**) of the reciprocal relaxation times of the particle concentration fluctuations (τ_1) versus the square of the wave vector and (**b**) the dependence of the translational diffusion coefficients (D_{t1}) on the concentration for the fast mode.

Table S5. Translation coefficient (D_{t1}) and radius of equivalent sphere (R_{h1}) corresponding to the fast mode.

c, %	D_{t1} *10[12], m[2]/c	St.D. D_{t1} *10[12], m[2]/c	R_{h2}, nm	St.D. R_{h2}, nm
0.0994	7.5	0.7	10	1
0.0796	8.2	0.9	9.1	1.0
0.0598	9.4	0.6	7.9	0.5
0.0397	8.2	0.6	9.0	0.7
**average value**	**8**	**1**	**9**	**1**

Table S6 shows Characteristic relaxation times of particle concentration fluctuations (τ_2) in the scattered volume (slow mode). Figure S11 shows plot of reciprocal relaxation times of particle concentration fluctuations (τ_2) versus the square of the wave vector and plot of translational diffusion coefficients (D_{t2}) versus concentration (right) for the slow mode. Table S7 shows Translational diffusion coefficient (D_{t2}) and hydrodynamic radius of equivalent sphere (R_{h2}) corresponding to the slow mode.

Table S6. Characteristic relaxation times of particle concentration fluctuations (τ_2) in the scattered volume (slow mode).

	C, %	0.0994	0.0796	0.0598	0.0397
**Θ, °**	**q^2 *10^{14}, 1/m²**	**τ_2, mc**			
40	1.843	-	12.6300	14.6900	12.6300
50	2.814	9.3300	8.0200	8.0200	8.0200
60	3.938	5.9260	5.9260	5.9260	5.6487
70	5.182	4.3790	4.3790	4.1743	3.9697
80	6.509	3.2360	3.2360	3.2360	2.9333
90	7.876	2.7820	3.0847	2.5213	2.5213
100	9.244	2.3910	2.2980	2.0560	2.0560
110	10.57	2.0560	2.0560	2.0560	-
120	11.81	1.7670	1.7670	1.7670	1.5190
130	12.94	1.7670	1.4480	1.3770	1.5190
140	13.91	1.4480	1.5190	1.3060	1.3060

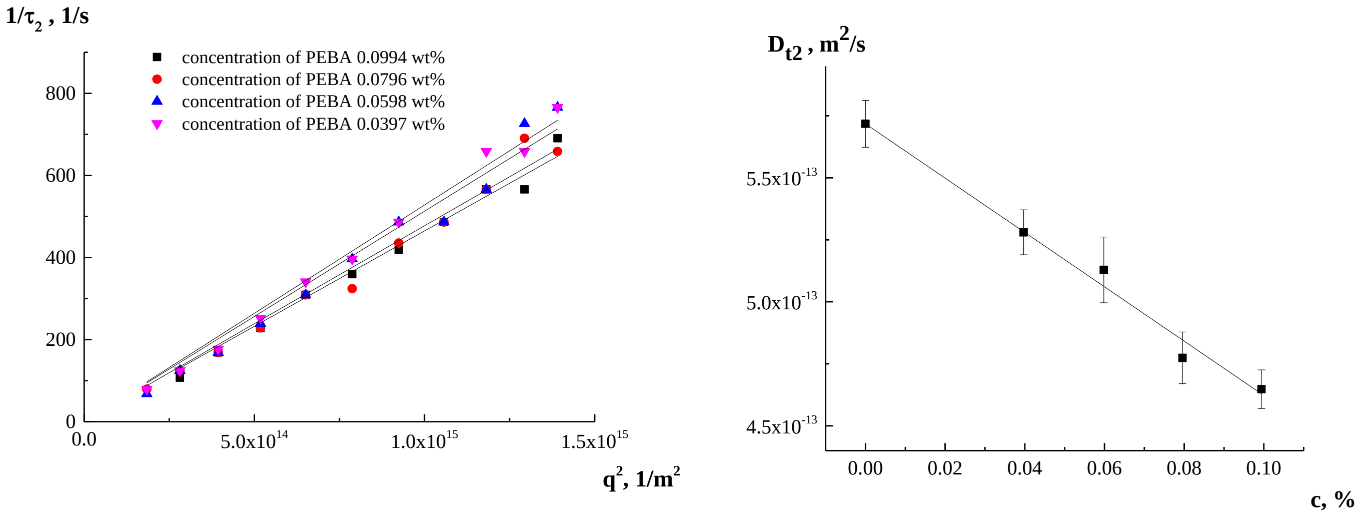

(**a**) (**b**)

Figure S11. (**a**) Plot of reciprocal relaxation times of particle concentration fluctuations (τ_2) versus the square of the wave vector and (**b**) plot of translational diffusion coefficients (D_{t2}) versus concentration (right) for the slow mode.

Table S7. Translational diffusion coefficient (D_{t2}) and hydrodynamic radius of equivalent sphere (R_{h2}) corresponding to the slow mode.

c, %	D_{t1} *10[12], m²/c	St.D. D_{t1} *10[12], m²/c	R_{h2}, nm	St.D. R_{h2}, nm
0.0994	0.46	0.01	160	3
0.0796	0.48	0.01	156	3
0.0598	0.51	0.01	145	4
0.0397	0.53	0.01	141	2
**Result of the extrapolation to the zero concentration**	**0.57**	**0.02**	**130**	**5**

Table S8 shows integrated intensity of the light scattering for polymer solutions and its standard deviation.

Table S8. Integrated intensity of the light scattering (I) for polymer solutions and its standard deviation (St.D. I).

**C, %**	**0.0994**	**0.0796**	**0.0598**	**0.0397**	**0.0994**	**0.0796**	**0.0598**	**0.0397**
**Θ, °**	**I**				**St.D. I, %**			
40	-	929429	697851	388315	-	11	12	11
50	625753	497455	372282	217004	8	9	9	8
60	389097	309174	229211	141521	6	6	7	5
70	274854	220204	167561	103073	5	5	5	5
80	209991	166008	129202	81302	4	5	4	4
90	171304	134837	106163	68558	4	3	4	3
100	147543	118916	93010	60937	3	3	3	2
110	135707	110010	84877	57494	2	2	2	2
120	132537	107357	83839	57418	2	2	3	2
130	137737	111989	88637	60667	2	2	2	2
140	152093	124289	98473	68475	2	2	2	1

Relative and reduced viscosity of PEBA were investigated at different temperature: at 10 °C (Table S9), at 20 °C (Table S10), at 25 °C (Table S11), at 30 °C (Table S12), at 40 °C (Table S13), at 50 °C (Table S14).

Table S9. Results of measurement of relative and reduced viscosity at 10 °C.

**c, g/dL**	**t, c**	**t/t_0**	**$(t/t_0-1)/c$, dL/g**	**$\ln(t/t_0)/c$, dL/g**
concentration	*rolling time of the ball in solution*	*relative viscosity*	*Huggins*	*Kramer*
0	92.77		-	
2	278.44	3.00	1.0007	0.5495
0.747	143.43	1.55	0.7311	0.5833
0.622	133.74	1.44	0.7100	0.5880
0.502	125.12	1.35	0.6946	0.5959
0.350	114.47	1.23	0.6685	0.6007

Table S10. Results of measurement of relative and reduced viscosity at 20 °C.

**c, g/dL**	**t, c**	**t/t_0**	**$(t/t_0-1)/c$, dL/g**	**$\ln(t/t_0)/c$, dL/g**
concentration	*rolling time of the ball in solution*	*relative viscosity*	*Huggins*	*Kramer*
0	70.02		-	
2	206.63	2.95	0.9754	0.5410
0.747	107.73	1.54	0.7209	0.5767
0.622	100.58	1.44	0.7016	0.5822
0.502	94.12	1.34	0.6855	0.5891
0.350	86.25	1.23	0.6623	0.5956

Table S11. Results of measurement of relative and reduced viscosity at 25 °C.

**c, g/dL**	**t, c**	**t/t_0**	**$(t/t_0-1)/c$, dL/g**	**$\ln(t/t_0)/c$, dL/g**
concentration	*rolling time of the ball in solution*	*relative viscosity*	*Huggins*	*Kramer*
0	61.28		-	
2	179.41	2.93	0.9639	0.5371
0.747	94.13	1.54	0.7176	0.5746
0.622	87.88	1.43	0.6978	0.5795
0.502	82.29	1.34	0.6828	0.5871
0.350	75.44	1.23	0.6602	0.5939

Table S12. Results of measurement of relative and reduced viscosity at 30 °C.

c, g/dL	t, c	t/t₀	(t/t₀-1)/c, dL/g	ln(t/t₀)/c, dL/g
concentration	*rolling time of the ball in solution*	*relative viscosity*	*Huggins*	*Kramer*
0	53.91		-	
2	156.63	2.91	0.9528	0.5333
0.747	82.54	1.53	0.7110	0.5703
0.622	77.19	1.43	0.6942	0.5771
0.502	72.29	1.34	0.6792	0.5844
0.350	66.30	1.23	0.6568	0.5912

Table S13. Results of measurement of relative and reduced viscosity at 40 °C.

c, g/dL	t, c	t/t₀	(t/t₀-1)/c, dL/g	ln(t/t₀)/c, dL/g
concentration	*rolling time of the ball in solution*	*relative viscosity*	*Huggins*	*Kramer*
0	42.19		-	
2	121.12	2.87	0.9355	0.5273
0.747	64.43	1.53	0.7059	0.5670
0.622	60.26	1.43	0.6887	0.5732
0.502	56.44	1.34	0.6732	0.5799
0.350	51.83	1.23	0.6529	0.5881

Table S14. Results of measurement of relative and reduced viscosity at 50 °C.

c, g/dL	t, c	t/t₀	(t/t₀-1)/c, dL/g	ln(t/t₀)/c, dL/g
concentration	*rolling time of the ball in solution*	*relative viscosity*	*Huggins*	*Kramer*
0	33.56		-	
2	95.23	2.84	0.9188	0.5215
0.747	51.06	1.52	0.6979	0.5617
0.622	47.79	1.42	0.6818	0.5684
0.502	44.80	1.33	0.6672	0.5754
0.350	41.17	1.23	0.6476	0.5837

S4. PEBA/Ho-MOFs Investigation

The surface of developed PEBA/Ho-MOFs membranes was studied using a light microscope. The optical micrographs for PEBA/Ho-MOFs membranes are presented in Figure S12.

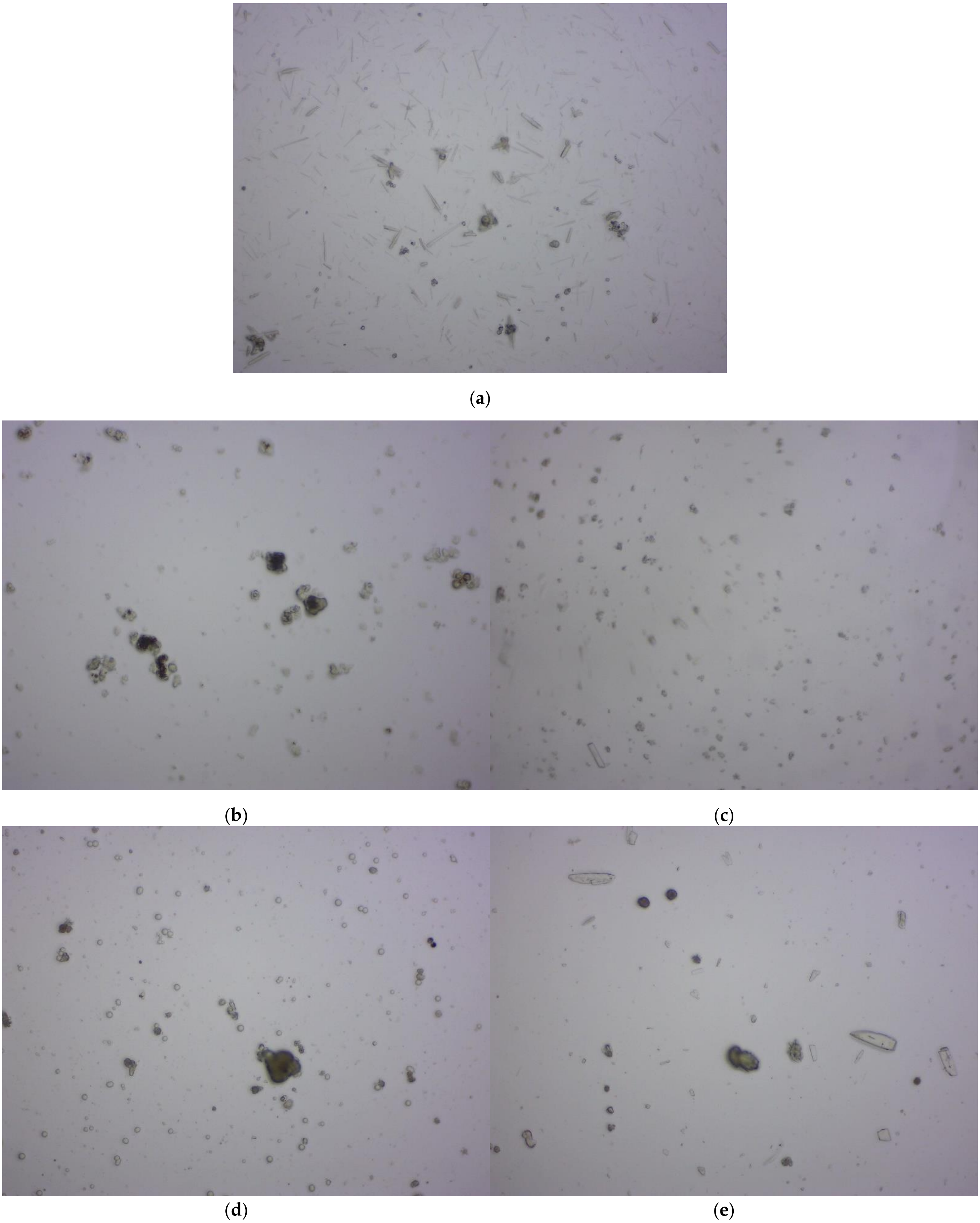

Figure S12. Optical micrographs for PEBA/Ho-MOFs membranes: (**a**) PEBA/Ho-1,3,5-H_3btc; (**b**) PEBA/Ho-1,2,4-H_3btc; (**c**) PEBA/Ho-1,2-H_2bdc; (**d**) PEBA/Ho-1,3-H_2bdc; (**e**) PEBA/Ho-1,4-H_2bdc.

References

1. Singha, D.K.; Majee, P.; Mondal, S.K.; Mahata, P. Detection of pesticide using the large stokes shift of luminescence of a mixed lanthanide co-doped metal–organic framework. *Polyhedron* **2019**, *158*, 277–282, doi:10.1016/j.poly.2018.10.066.

2. Fröhlich, D.; Pantatosaki, E.; Kolokathis, P.D.; Markey, K.; Reinsch, H.; Baumgartner, M.; van der Veen, M.A.; De Vos, D.E.; Stock, N.; Papadopoulos, G.K.; et al. Water adsorption behaviour of CAU-10-H: a thorough investigation of its structure–property relationships. *J. Mater. Chem. A* **2016**, *4*, 11859–11869, doi:10.1039/C6TA01757F.

3. Almáši, M.; Zeleňák, V.; Kuchár, J.; Bourrelly, S.; Llewellyn, P.L. New members of MOF-76 family containing Ho(III) and Tm(III) ions: Characterization, stability and gas adsorption properties. *Colloids Surfaces A Physicochem. Eng. Asp.* **2016**, *496*, 114–124, doi:10.1016/j.colsurfa.2015.10.048.
